# Supplementary material for: Cloning southern corn rust resistant gene RppK and its cognate gene AvrRppK from Puccinia polysora
Source: Nat Commun. 2022 Jul 29;13:4392. doi: 10.1038/s41467-022-32026-4 (PMC9338322; doi:10.1038/s41467-022-32026-4)
Supplement: Supplementary file 1 — Supplementary Information [file 41467_2022_32026_MOESM1_ESM.pdf]

Cloning southern corn rust resistant gene *RppK* and its cognate gene *AvrRppK* from *Puccinia polysora*

Chen *et al.*

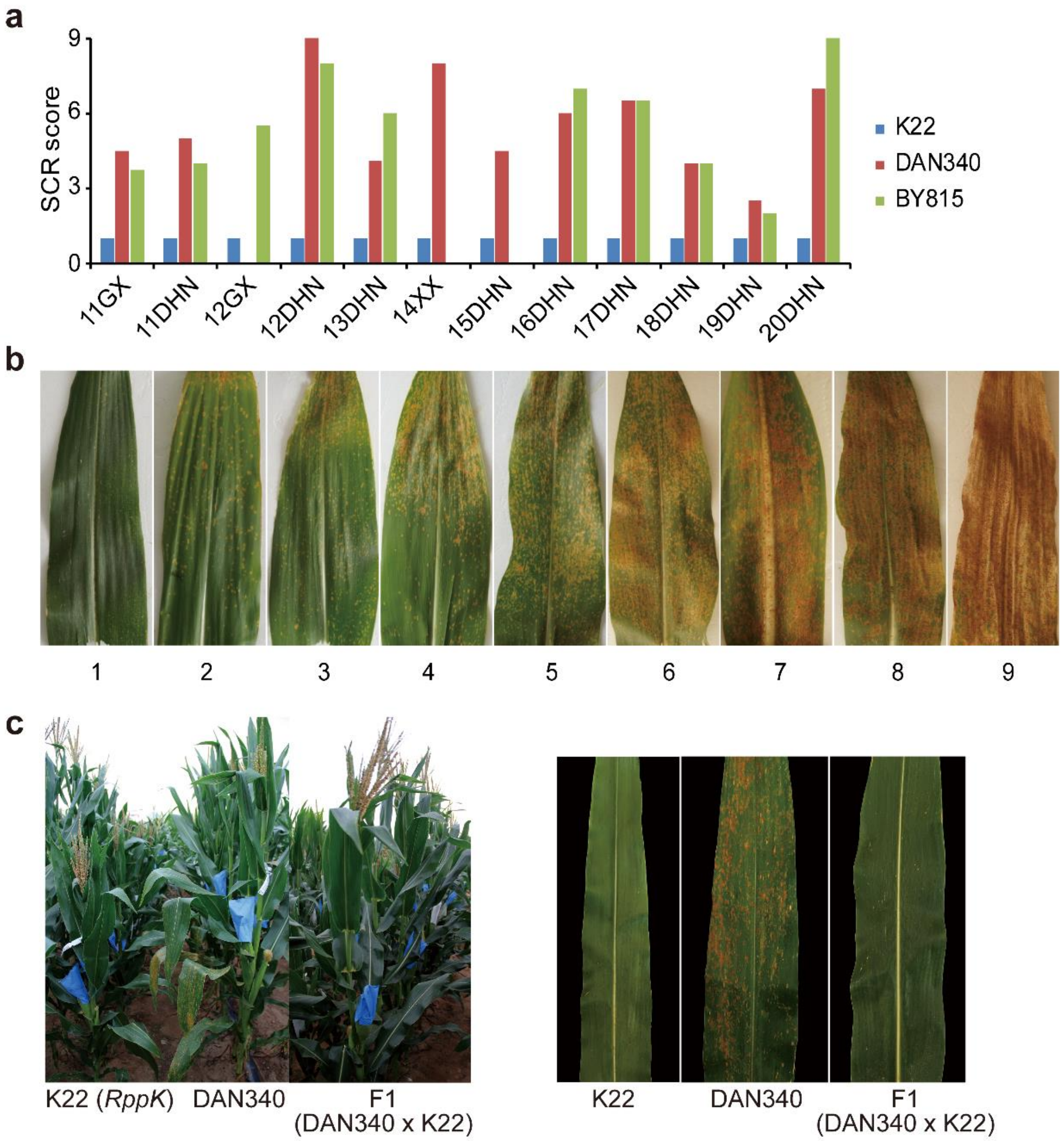

Supplementary Figure 1. The SCR disease scale and SCR disease phenotypes of inbred lines K22, DAN340 and F_1 (DAN340 × K22). (a). The field SCR scores from 2011 to 2020 indicated that K22 showed durable resistance to *P. polysora* for approximately ten years. In 11GX, the number represents the year, and GX represents Nanning, Guangxi Province. DHN represents Sanya, Hainan Province. XX represents Xinxiang, Henan Province. K22 is resistant to SCR and DAN340 is susceptible to SCR. BY815, a very susceptible inbred line to SCR, was taken as a control. **(b)** The SCR disease scale ranges from 1 to 9, where "1" indicates complete resistance, with no disease symptoms or only an HR on leaves, and "9" indicates infected pustules over entire leaves and death of the plants. **(c)** SCR disease phenotypes of K22, DAN340 and F_1 (DAN340 × K22) plants. Source data are provided as a Source Data file.

Marker	DAN340	Key recombinants 1	2	3	4	5	6	7	8	9	10	11	12	13	14	15	16	17	K22
RRD39	D	D	K	D	K	D	K	D	D	D	K	K	D	K	K	D	K	D	K
RRD40	D	D	D	D	K	D	K	D	D	D	K	K	D	K	K	D	K	D	K
InDel7-3	D	D	D	K	D	D	K	D	D	D	K	K	D	K	K	D	K	D	K
SNP20	D	D	D	K	D	D	K	D	D	D	K	K	D	K	K	D	K	D	K
RRD103	D	D	D	K	D	K	D	D	D	D	K	K	D	K	K	D	K	D	K
RRD111	D	D	D	K	D	K	D	K	K	D	D	K	D	K	K	D	K	D	K
SNP5	D	D	D	K	D	K	D	K	K	K	D	K	D	K	K	D	K	D	K
InDel213	D	D	D	K	D	K	D	K	K	K	D	K	D	K	K	D	K	D	K
SSR258	D	D	D	K	D	K	D	K	K	K	D	D	D	K	K	D	K	D	K
InDel262	D	D	D	K	D	K	D	K	K	K	D	D	D	K	K	D	K	D	K
RRD64	D	D	D	K	D	K	D	K	K	K	D	D	K	D	K	D	K	D	K
RRD26	D	D	D	K	D	K	D	K	K	K	D	D	K	D	D	K	K	D	K
SSR4	D	D	D	K	D	K	D	K	K	K	D	D	K	D	D	K	D	K	K
Phenotype	S	S	S	R	S	R	S	R	S	S	S	R	S	R	R	S	R	S	R

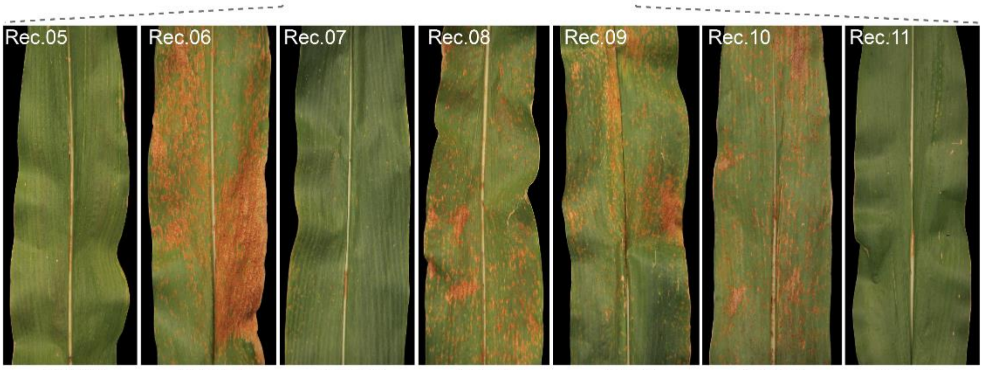

4H1213 4H1169 4H1083 4H1475 4H943 4H1652 4H1225

Supplementary Figure 2. Genotypes and phenotypes of the key recombinant lines used for the fine-mapping of *RppK*. In the upper table, “K” indicates the genotype of the K22 allele; “D” indicates the genotype of the DAN340 allele. R, resistant phenotype; S, susceptible phenotype. Rec: recombinant line. The SCR phenotypes of recombinants 5, 6, 7, 8, 9, 10, and 11 with family names are presented below. The primers of the markers are listed in Supplementary Data 5.

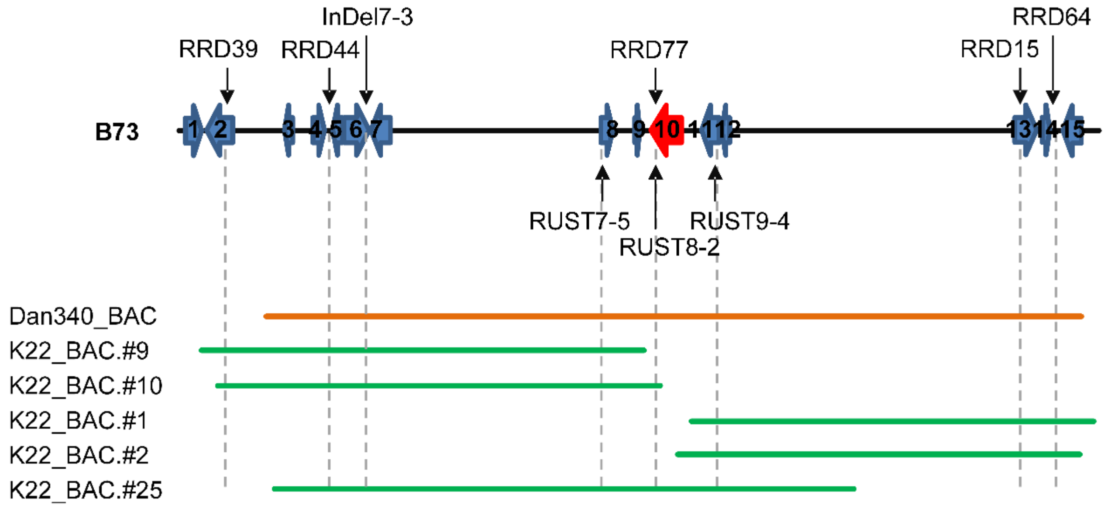

Supplementary Figure 3. BAC clones covering the *RppK* locus. The locations of genes (labelled from 1 to 15) at the *RppK* locus are indicated by colored arrows based on the B73 reference genome, and the red arrow (gene 10) indicates the *Zm00001d023267* gene. Two BAC libraries were generated by using genomic DNA isolated from two maize inbred lines (K22 and DAN340). Three markers (RUST7-5, RUST8-2 and RUST9-4) were used to screen positive BAC clones, and six markers (RRD39, RRD44, InDel7-3, RRD77, RRD15 and RRD64) were used to validate the overlapping regions between the BACs. Marker sequences are listed in Supplementary Data 5. The orange line represents the DAN340 BAC clone. The green lines represent five K22 BAC clones. All BAC clones were sequenced by PacBio sequencing.

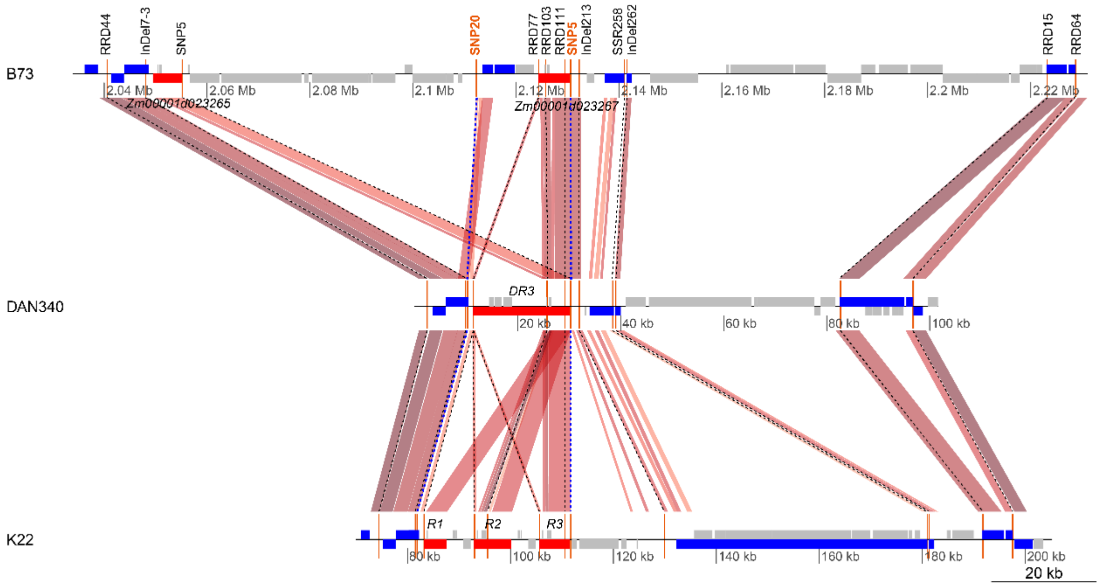

Supplementary Figure 4. Genomic variation of *RppK* regions among B73, two parental lines DAN340 and K22. The blue and red rectangles represent genes, and the gray rectangles represent transposon elements. The rectangles above the (+1) or below the (-1) line represent the transcription orientation. The red rectangles represent *NLR* genes (*Zm00001d023265* and *Zm00001d023267* in B73; *DR3* in DAN340; *R1*, *R2* and *R3* in K22). Light-red shading indicates collinear regions. The locations of markers in B73, DAN340 and K22 are indicated by vertical orange lines and are connected with dotted lines between the three different maize genomes. The region between the blue dotted lines is the *RppK* region, which is flanked by markers SNP20 and SNP5 (highlighted in orange color).

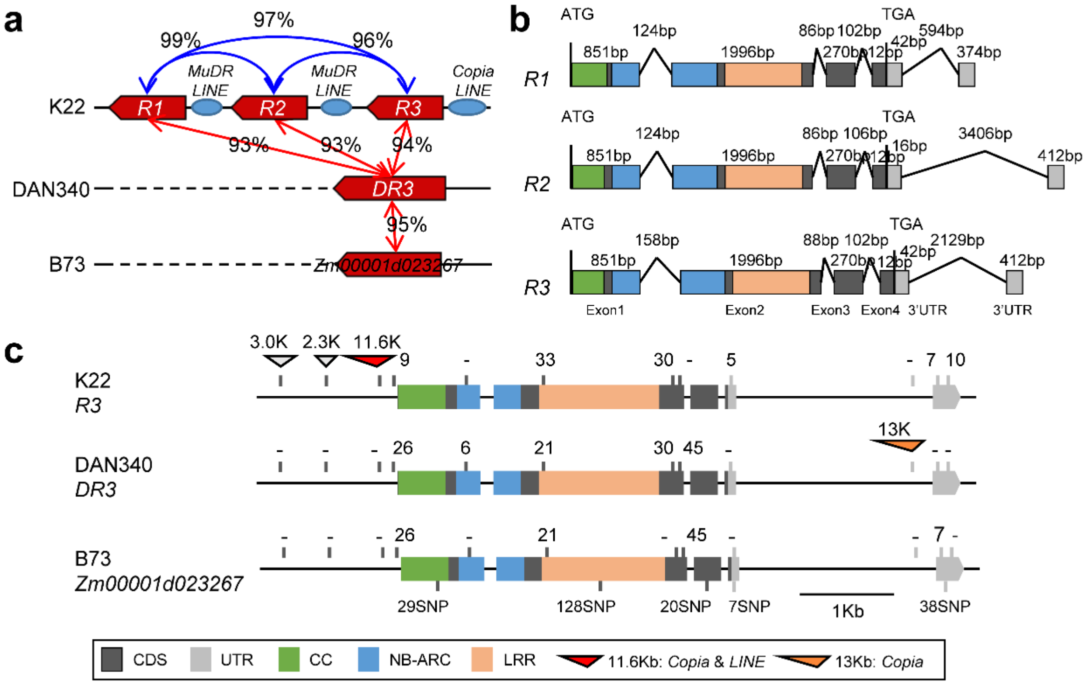

Supplementary Figure 5. Gene variation at the *RppK* locus among K22, DAN340 and B73. (a) Comparison of the *RppK* loci of the K22, DAN340 and B73 lines. The red boxed arrows indicate genes and transcription orientation, while the blue ovals indicate transposons. The nucleotide identities of the *R1*, *R2* and *R3* genes are indicated with blue double-sided arrows, while the nucleotide identities of *NLRs* among K22 (*R1*, *R2* and *R3* genes), DAN340 (*DR3* gene) and B73 (*Zm00001d023267*) are indicated with red double-sided arrows. **(b)** Gene structures of three *NLR* genes at the *RppK* locus. The full-length open reading frame (ORF) of *R3* was determined by a RACE assay and sequencing using the primers listed in Supplementary Data 5. The ORFs of the *R1* and *R2* genes were predicted based on the *R3* cDNA sequence. The 3'-untranslated regions (UTRs) are indicated by gray rectangles. Exons are indicated by dark gray rectangles. Introns are indicated by lines. The positions of the coding regions of the CC, NB-ARC and LRR domains are indicated by dark green, blue and orange rectangles, respectively. The start codons (ATG) and stop codons (TGA) are indicated by vertical black lines. **(c)** The variation in the promoter and coding sequences of the *R3* gene among K22, DAN340 and B73. Inverted triangles indicate fragment insertions, and the red and orange inverted triangles represent large insertions containing transposons (e.g., *Copia* and *LINE*). The different base lengths of deletion/insertion variation sites are indicated with vertical lines above the genes, and dashed lines represent deletions. The numbers of SNPs in each exon are shown below the genes. The coding sequences are indicated by dark gray rectangles. The 3'-UTRs are indicated by gray rectangles. The positions of the coding regions of the CC, NB-ARC and LRR domains are indicated by dark green, blue, and orange rectangles respectively.

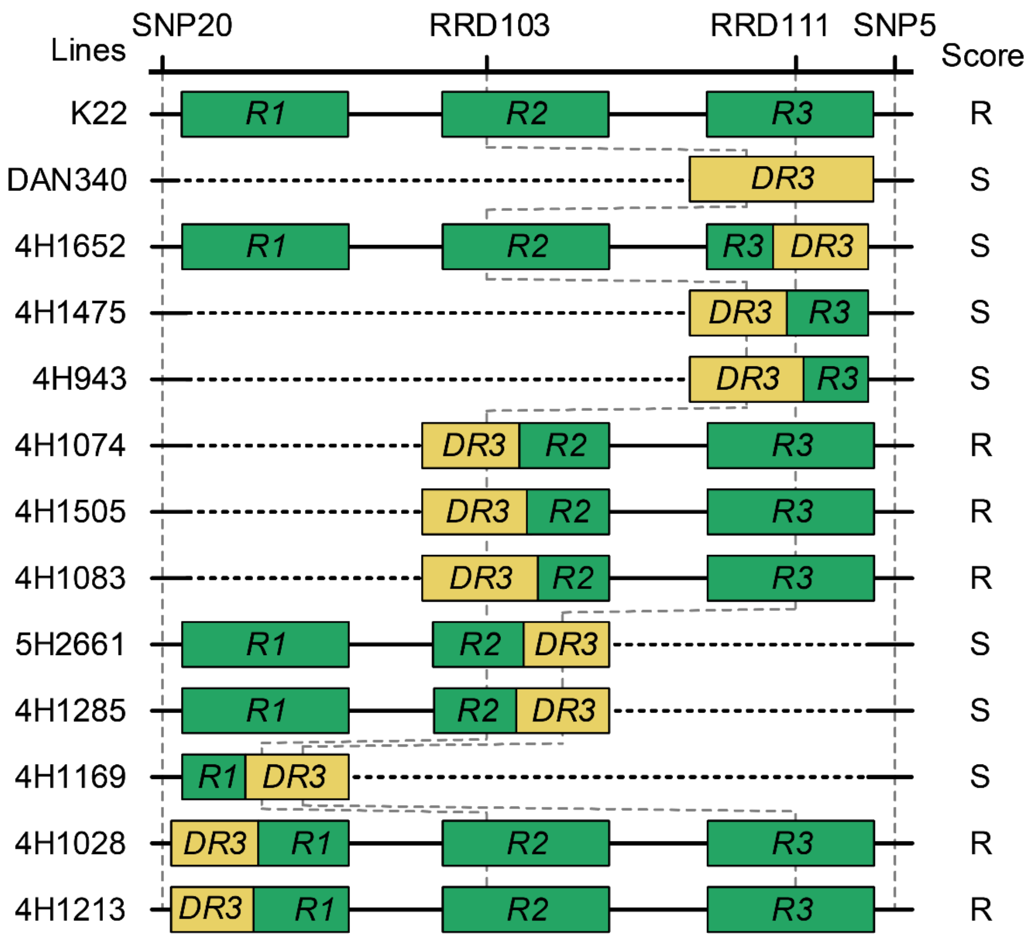

Supplementary Figure 6. Schematic representation of the gene structure of the *RppK* locus in eleven key recombinants. Green segments represent the K22 allele, and yellow segments represent the DAN340 allele. The locations of four markers, SNP20, RRD103, RRD111 and SNP5, are shown above and are connected with dashed gray lines between the recombinants. The dotted lines in DAN340 and the recombinants represent deletions, compared with the genomic sequence of K22. R: resistant phenotype, S: susceptible phenotype. *R1*, *R2* and *R3* are three *NLR* genes in K22. *DR3*: *DAN340 R gene homologous to R3*.

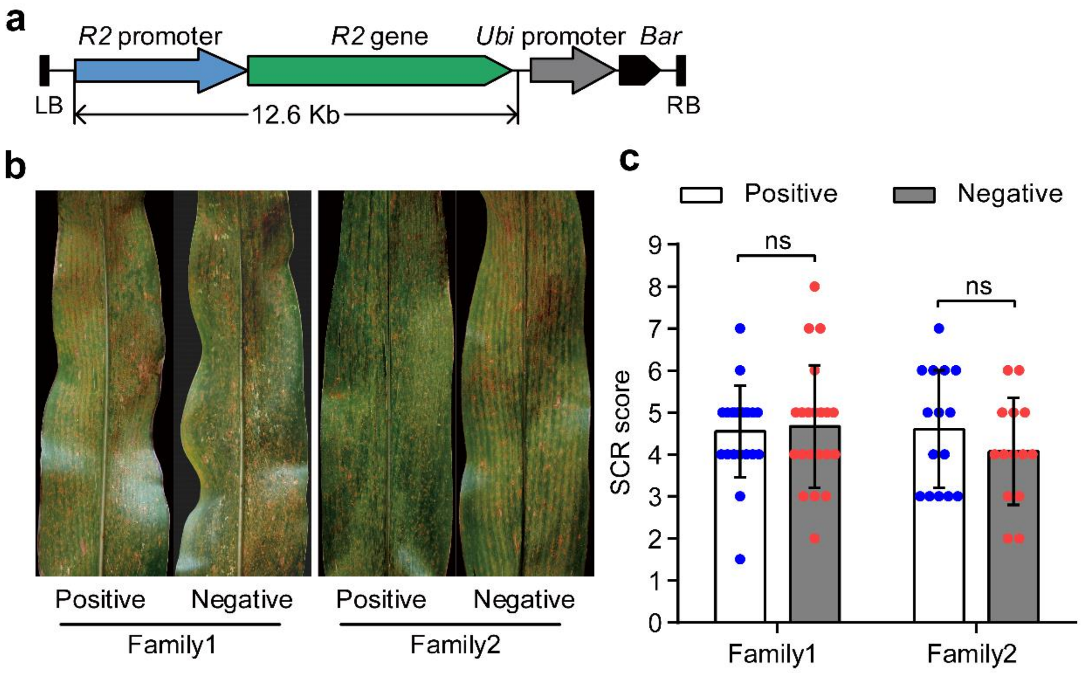

Supplementary Figure 7. SCR disease phenotypes of *R2* transgenic plants. (a) The structure of the *R2* genomic sequence construct used for transformation of the susceptible line KN5585. LB, left border; RB, right border; *Ubi*: *ZmUbiquitin* gene; *Bar*: *Bialaphos Resistance* Gene. The construct contains the entire *R2* genomic DNA sequence, including the 4.7 kb promoter region and the 4.4 kb downstream sequence. **(b)** *R2*-positive transgenic plants of two independent lines were susceptible to SCR, similar to *R2*-negative transgenic plants. **(c)** The disease scales of *R2* transgenic plants of two independent families. Values are means ± SDs (Student's *t* test, two-tailed), n= 21, 21, 15, and 14 individual plants of the positive and negative lines of family 1 and family 2, respectively. ns, not significant. Source data are provided as a Source Data file.

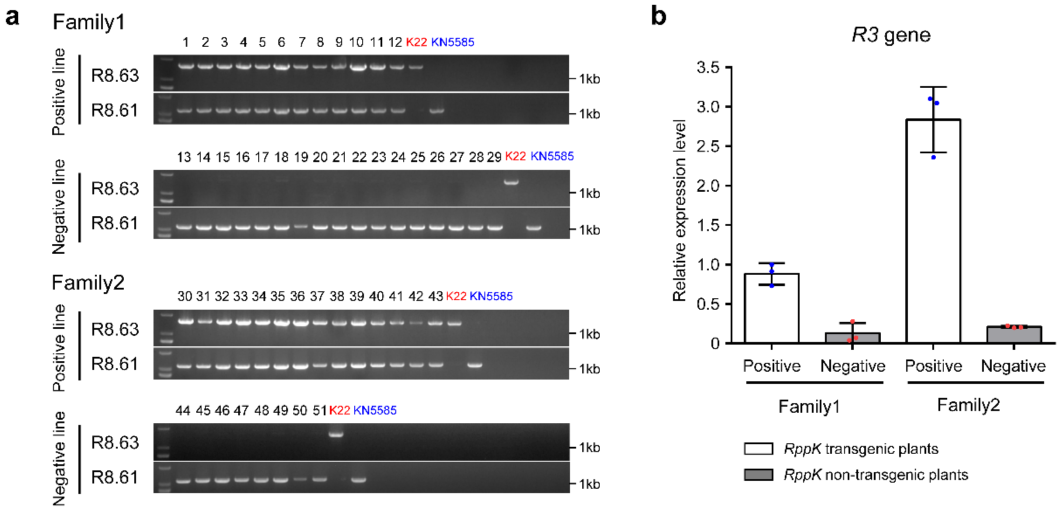

Supplementary Figure 8. Evaluation of *RppK* transgenic families. (a) Identification of positive and negative plants in two *RppK* transgenic families by using molecular markers (R8.63 and R8.61). K22 was taken as the positive control and KN5585 was taken as the negative control. R8.63 is specific to *R3* gene; a specific PCR fragment was amplified from *RppK* transgenic positive plants and K22 by using R8.63 and no PCR fragment was amplified from *RppK* transgenic negative plants. R8.61 is specific to *DR3* gene which exists in Dan340 and KN5585, but not in K22. So, a specific PCR fragment was amplified from all transgenic positive and negative plants, but not in K22. Here, R8.61 was used to test the DNA quality. **(b)** Detection of *RppK* gene expression in two *RppK* transgenic families by RT-qPCR. Values are means ± SDs, n = 3 samples. Source data are provided as a Source Data file.

a

P. polysora isolate	*AvrRppC* genotype	*AvrRppK* genotype
PP.CN1.0	$AvrRppC^{ref}$ + $AvrRppC^{E}$	$AvrRppK^{ref}$
PP.CN2.0	$AvrRppC^{C}$ + $AvrRppC^{F}$	$AvrRppK^{ref}$
PP.CN3.0	$AvrRppC^{A}$ + $AvrRppC^{J}$	$AvrRppK^{ref}$
PP.Hainan	$AvrRppC^{ref}$ + $AvrRppC^{E}$	$AvrRppK^{ref}$
PP.Guangdong	$AvrRppC^{J}$ + $AvrRppC^{L}$	$AvrRppK^{ref}$
PP.Wuhan	$AvrRppC^{E}$+ $AvrRppC^{L}$	$AvrRppK^{ref}$
PP.Guangxi	$AvrRppC^{ref}$ + $AvrRppC^{E}$	$AvrRppK^{ref}$

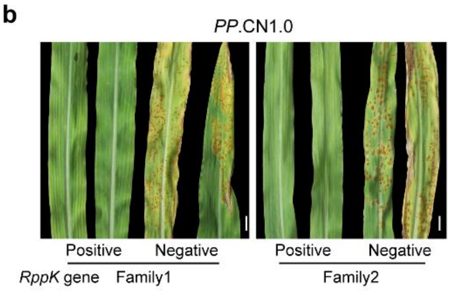

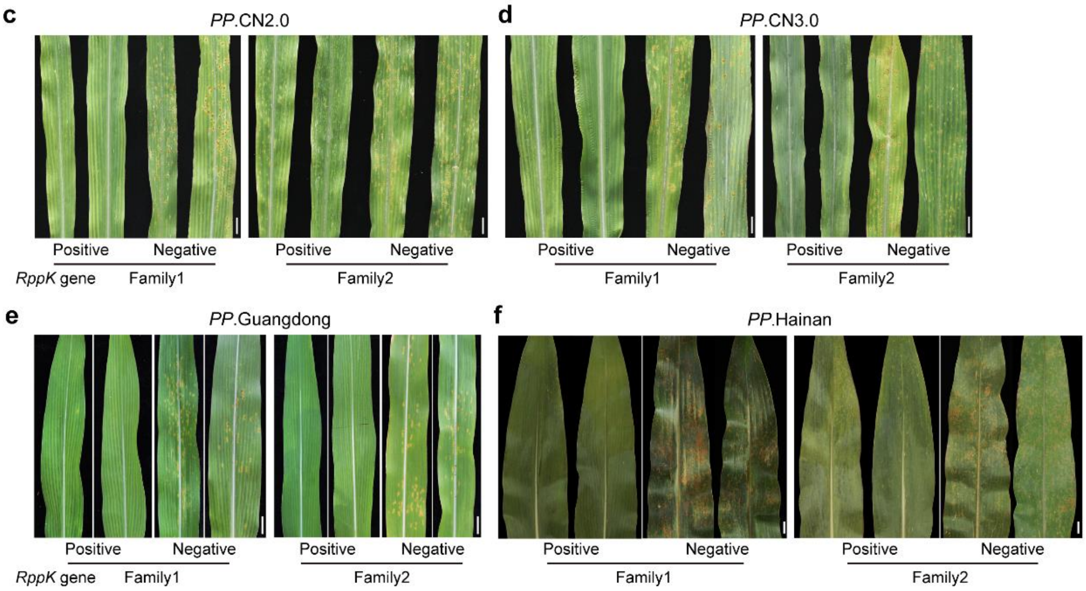

Supplementary Figure 9. Examination of the resistance phenotypes of *RppK* transgenic plants to five *P. polysora* isolates. (a) Genotypes of *AvrRppC* and *AvrRppK* in seven *P. polysora* isolates. **(b)** The disease phenotype of two *RppK* transgenic families to isolate *PP*.CN1.0. **(c)** The disease phenotype of two *RppK* transgenic families to isolate *PP*.CN2.0. **(d)** The disease phenotype of two *RppK* transgenic families to isolate *PP*.CN3.0. **(e)** The disease phenotype of two *RppK* transgenic families to isolate *PP*.Guangdong. **(f)** The disease phenotype of two *RppK* transgenic families to isolate *PP*.Hainan.

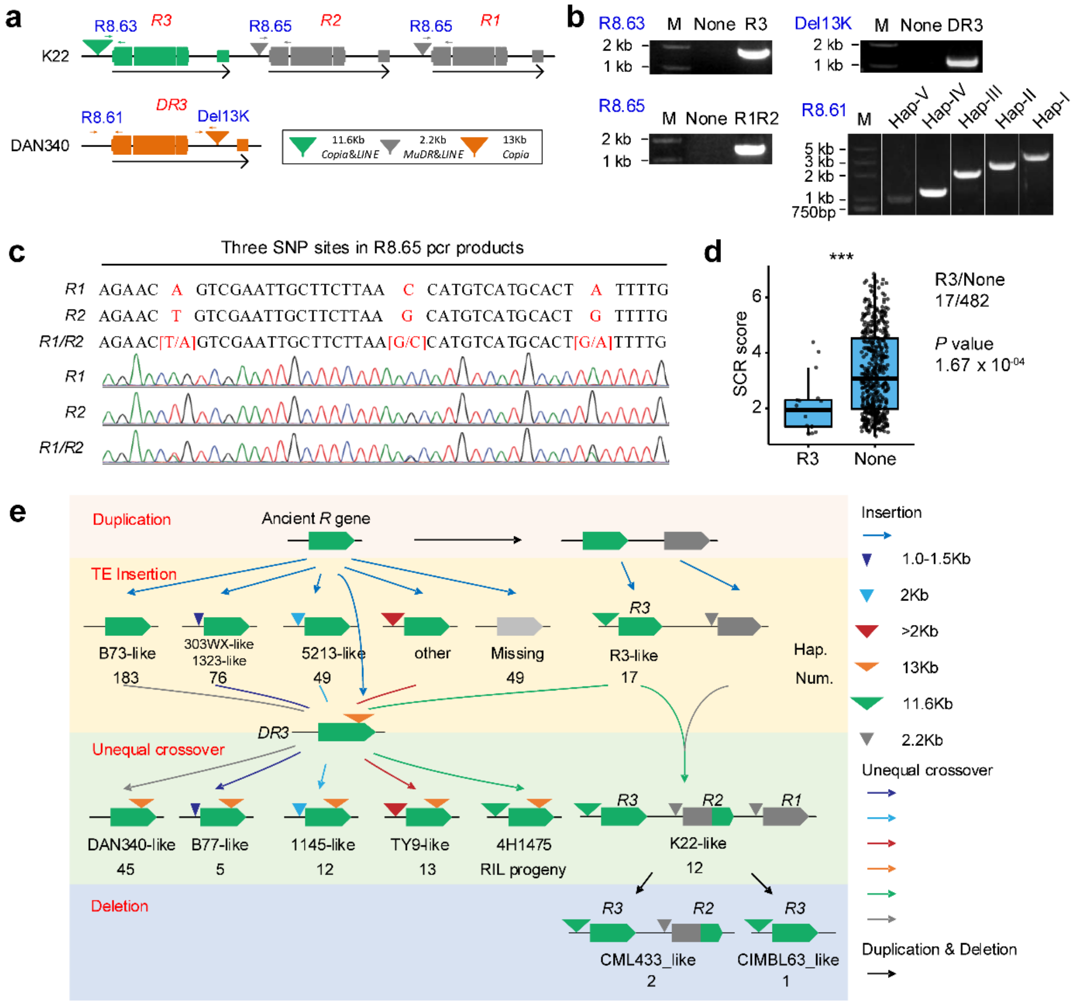

Supplementary Figure 10. Detection of the *RppK* haplotype and its evolutionary roadmap. (a) The regions amplified by targeting four markers, R8.63, R8.65, R8.61 and Del13K. The relative locations of the primers are shown above the corresponding genes. The R8.63, R8.65 and Del13K primers were used to detecting transposon insertions (inverted triangles). The R8.61 primer pair specifically amplified the promoter region and partial exon 1 region of *DR3*. The R8.65 primer pair was used to amplify promoter sequences of the *R1* gene and *R2* gene of the same size, and there were only three ACA/TGG polymorphisms in the R8.65 products between the *R1* and *R2* genes shown in Supplementary Fig. 10c. **(b)** Gel images of the four markers, R8.63, R8.65, R8.61 and Del13K. Five different sizes of R8.61 products were detected in the association mapping panel. "None", no PCR product amplified; "M", marker. The experiment was repeated three times with similar results. This experiment was repeated three times with similar results. **(c)** The *R1* and *R2* fragments were distinguished by sequencing the R8.65 PCR products. The ACA haplotype represents the *R1* fragment, and the TGG haplotype represents the *R2* fragment. **(d)** ANOVA of SCR resistance between lines carrying the *R3* gene and those without the *R3* gene. The lines containing the *R3* gene (n=17) were significantly more resistant to SCR than the non-*R3*-carrying lines (n=482). In box plots, the center line represents the median, box edges delimit

upper and lower quartiles, and the whiskers extend to maximum and minimum values excluding outliers. ***: $P<0.001$ (Student's *t*-test, two-tailed). **(e)** The evolutionary roadmap of the *RppK* gene. The evolution of the *RppK* locus involved a series of events, including transposon insertion, unequal crossover, duplication and deletion events. The classes of haplotypes and corresponding numbers are shown under the gene models. The boxed arrows represent *NLR* genes, and the inverted triangles with different colors and sizes represent the insertion fragments. The linear arrows represent unequal crossover events. Source data are provided as a Source Data file.

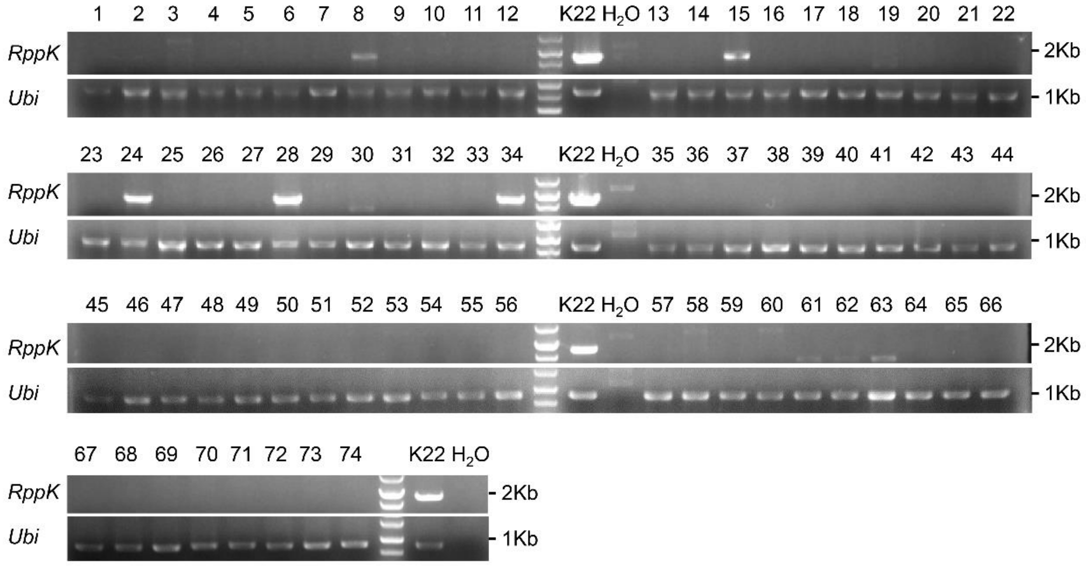

Supplementary Figure 11. Genotyping 74 maize hybrid cultivars. 74 hybrids commercially cultivated in China were genotyped by using molecular marker R8.63 to check whether they contain *RppK* gene. K22 was taken as the positive control in this assay. Maize *ubiquitin* gene was amplified to confirm that the quality of DNA samples was good. The experiment was repeated three times with similar results. Source data are provided as a Source Data file.

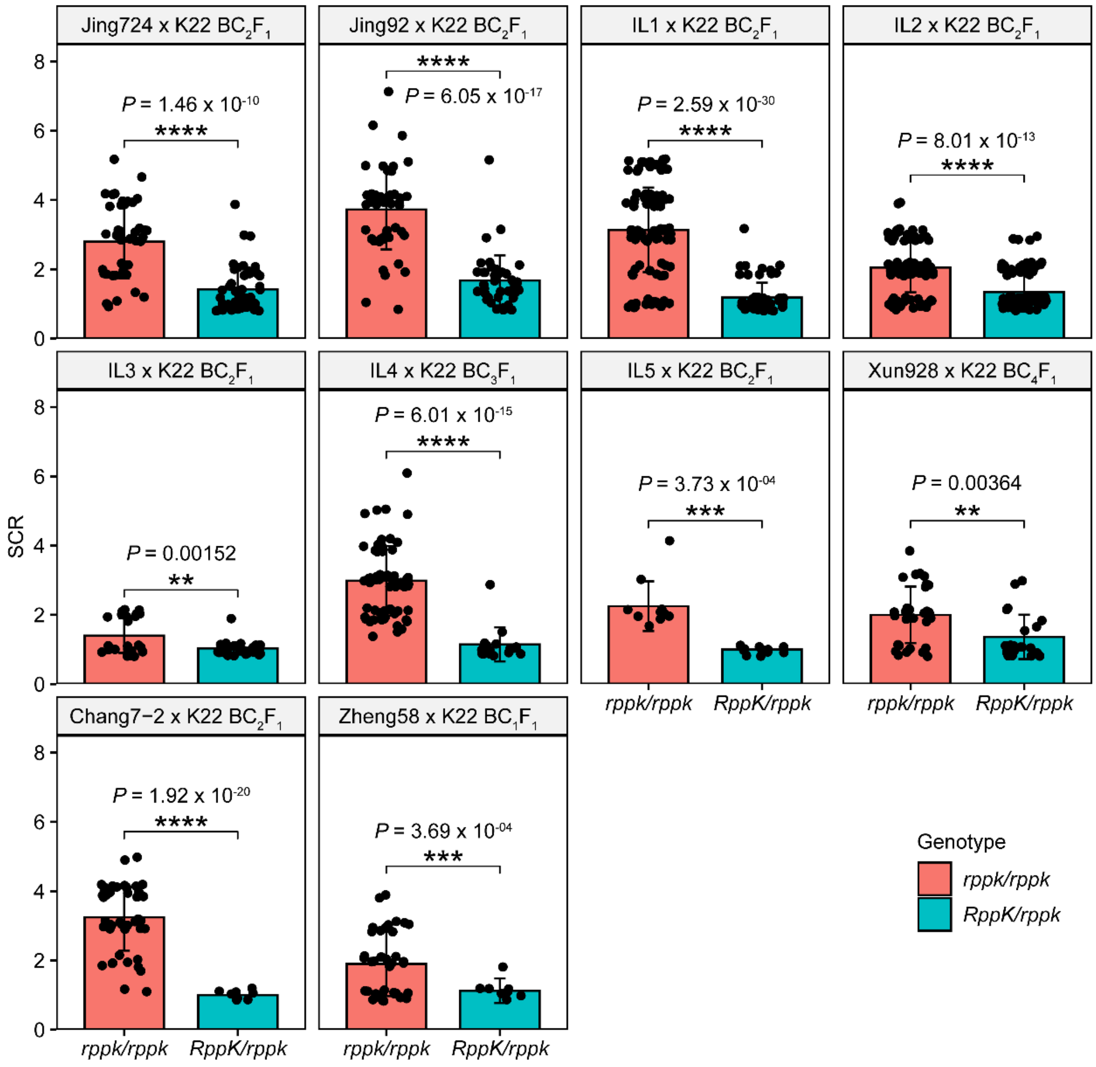

Supplementary Figure 12. The *RppK* locus improves maize resistance to SCR in different genetic backgrounds. The *RppK* locus was introgressed into ten different maize inbred lines (Jing724, Jing92, IL1, IL2, IL3, IL4, IL5, Xun928, Chang7-2 and Zheng58) via repeated backcrossing. Plants with the *RppK* locus were more resistant to SCR than plants without the *RppK* locus in the ten different maize genetic backgrounds under field conditions. *RppK/rppk* (n=50, 42, 65, 93, 31, 17, 11, 22, 8, 8) indicates that lines carry the *RppK* locus with a heterozygous genotype. *rppk/rppk* (n=44, 51, 103, 101, 25, 68, 10, 31, 47, 40) indicates that lines do not contain the *RppK* locus. Values are means ± SDs. **: P<0.01, ***: P<0.001, ****: P<0.0001 (Student's t-test, two-tailed). Source data are provided as a Source Data file.

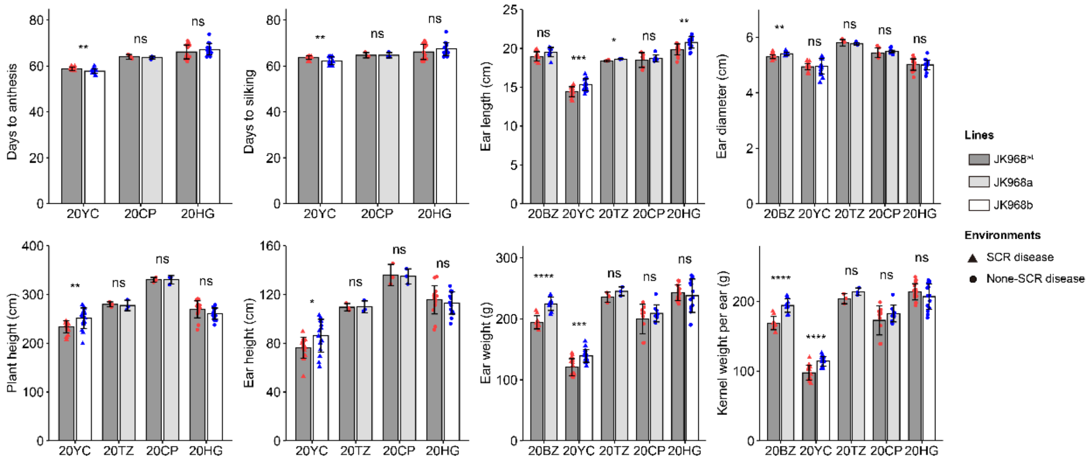

Supplementary Figure 13. The introgression of the *RppK* locus into hybrid JK968 does not affect its agronomic traits. Comparison of days to anthesis, days to silking, plant height, ear height, ear length, ear diameter, ear weight and kernel weight per ear between hybrids JK968^{wt} and JK968a or JK968b in the presence or absence of SCR disease. Field trials in the presence of SCR disease were conducted in Bozhou (BZ), with 9 repeat plots (38 plants of each line in each plot) and Yongcheng (YC) with 16 repeat plots (11 plants of each line in each plot), China, in 2020. Field trials in the absence of SCR disease were conducted in Tongzhou (TZ) with 3 repeat plots (38 plants of each line in each plot), Changping (CP) with 9 repeat plots (38 plants of each line in each plot) and Huanggang (HG) with 16 repeat plots (11 plants of each line in each plot), in China, in 2020. Values are means ± SDs. *P<0.05. **P<0.01. ***P<0.001. ****P<0.0001 (Student's t-test, two-tailed). ns, not significant. P values are presented in Source Data file. Source data are provided as a Source Data file.

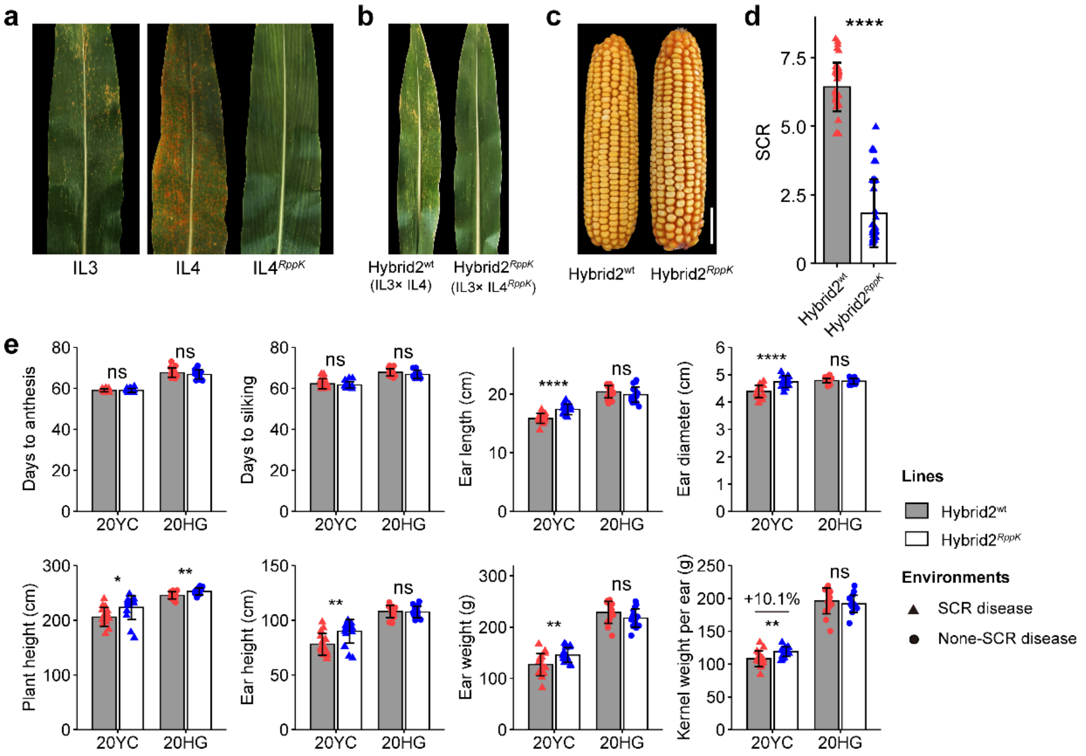

Supplementary Figure 14. The introgression of the *RppK* locus into Hybrid2 enhances its resistance against SCR and increases its grain yield under SCR disease pressure. **(a)** SCR disease phenotypes of wild type lines IL3, and IL4 and improved line IL4^{RppK} carrying the *RppK* allele in the field. **(b-c)** SCR disease phenotypes **(b)** and ears **(c)** of Hybrid2^{wt} and Hybrid2^{RppK} evaluated in the field in the presence of SCR disease. **(d)** SCR disease phenotypes were evaluated in Hybrid2^{wt} and improved Hybrid2^{RppK} carrying the *RppK* allele in Yongcheng, China in 2020. ****, *P*<0.0001 (Student's *t*-test, two-tailed). **(e)** Comparison of days to anthesis, days to silking, plant height, ear height, ear length, ear diameter, ear weight and kernel weight per ear between Hybrid2^{wt} and Hybrid2^{RppK} in the presence or absence of SCR disease. Field trials in the presence of SCR disease were conducted in Yongcheng (YC), with 16 repeat plots (11 plants of each line in each plot), in China, in 2020. Field trials in the absence of SCR disease were conducted in Huanggang (HG) with 16 repeat plots (11 plants of each line in each plot), China, in 2020. Values are means ± SDs. *P<0.05. **P<0.01. ****P<0.0001 (Student's *t*-test, two-tailed). ns, not significant. Source data are provided as a Source Data file.

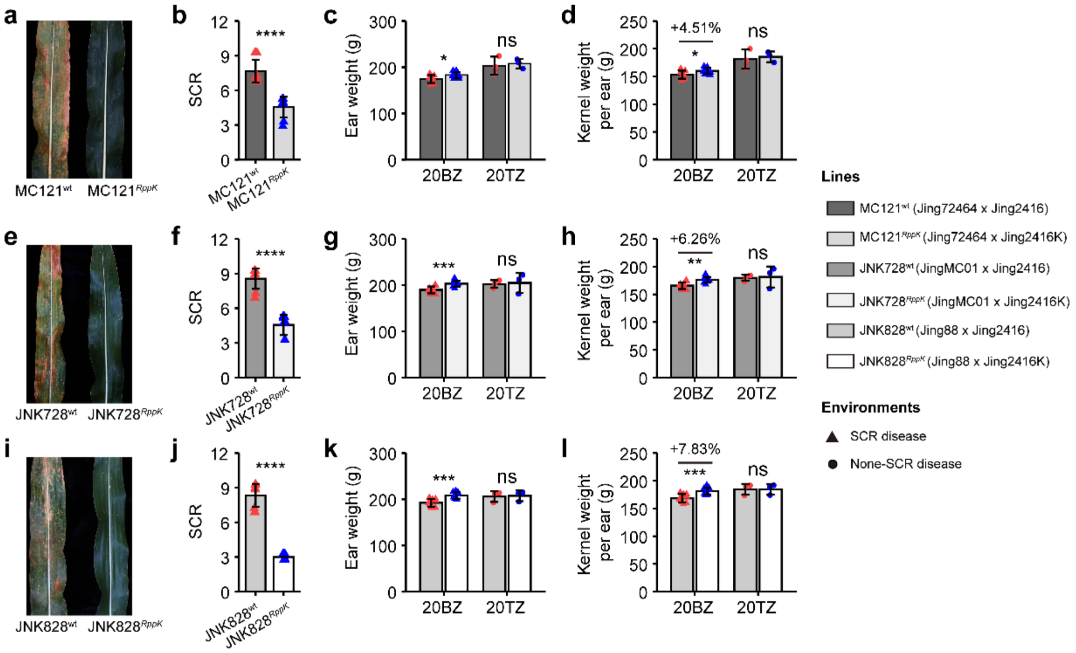

Supplementary Figure 15. The introgression of the *RppK* locus into hybrids (MC121^{wt}, JNK728^{wt} and JNK828^{wt}) enhances their resistance to SCR and increases their grain yield under SCR disease pressure. Hybrids (MC121^{wt}, JNK728^{wt} and JNK828^{wt}) were generated by crossing inbred lines, Jing72464, JingMC01 and Jing88, with Jing2416, respectively; and improved hybrids (MC121^{RppK}, JNK728^{RppK} and JNK828^{RppK}) were generated by respectively crossing inbred lines (Jing72464, JingMC01 and Jing88) with Jing2416^{RppK} which carries the *RppK* locus. **(a, e, i)** SCR disease phenotypes of MC121^{wt} and MC121^{RppK} **(a)**, JNK728^{wt} and JNK728^{RppK} **(e)**, and JNK828^{wt} and JNK828^{RppK} **(i)** evaluated in the field in the presence of SCR disease. **(b-d)** Comparisons of SCR resistance **(b)**, ear weight **(c)** and kernel weight per ear **(d)** in MC121^{wt} and MC121^{RppK} hybrids derived from crosses between inbred lines Jing72464 and Jing2416 or Jing2416K carrying the *RppK* locus. **(f-h)** Comparisons of the SCR resistance **(f)**, ear weight **(g)** and kernel weight per ear (**h**) in hybrids JNK728^{wt} and JNK828^{RppK} derived from crosses between inbred lines JingMC01 and Jing2416 or Jing2416K carrying the *RppK* locus. **(j-l)** Comparisons of SCR resistance **(j)**, ear weight **(k)** and kernel weight per ear **(l)** in hybrids JNK828^{wt} and JNK828^{RppK} derived from crosses between inbred lines Jing88 and Jing2416 or Jing2416K carrying the *RppK* locus. Field trials in the presence of SCR disease pressure were conducted with 9 repeat plots (38 plants of each line in each plot) in Bozhou, China in 2020. Field trials in the absence of SCR disease pressure were conducted with 3 repeat plots (38 plants of each line in each plot) in Tongzhou, China in 2020. Values are means±SDs. ns, not significant, **P*<0.05, ***P*<0.01, ****P*<0.001, *****P*<0.0001 (Student's *t*-test, two-tailed). Source data are provided as a Source Data file.

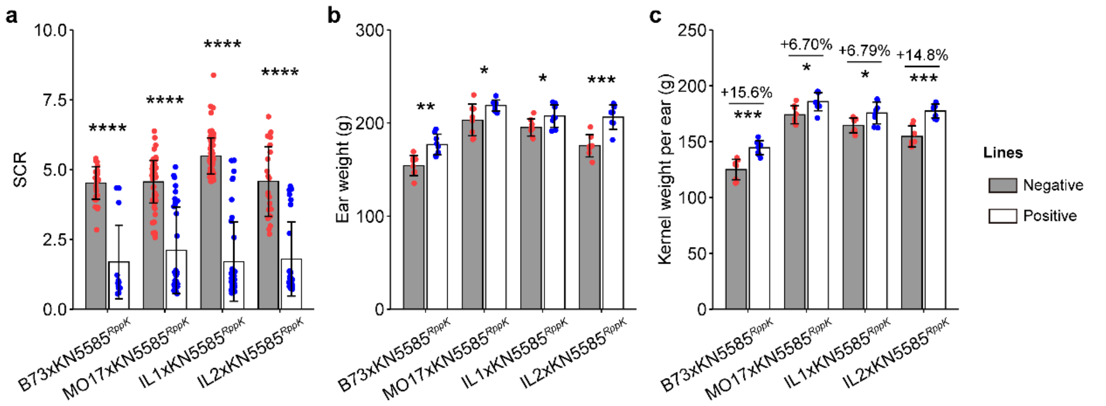

Supplementary Figure 16. The transgenic *RppK* gene enhances the resistance of maize hybrids to SCR and increases grain yields in four different hybrids in the presence of SCR disease. (a-c) Comparisons of SCR resistance **(a)**, ear weight **(b)** and kernel weight per ear **(c)** in four paired hybrids derived from crosses between inbred lines and KN5585^{RppK} or nontransgenic KN5585. Field trials in the presence of SCR disease pressure were conducted in Sanya, China in 2019. "Positive", hybrids derived from a cross between inbred lines and KN5585^{RppK}. "Negative", hybrids derived from a cross between inbred lines and nontransgenic KN5585. Values are the means±SDs, n= 8 repeat plots (11 plants of each line in each plot) in **(b)** and **(c)**, *P<0.05, **P<0.01, ***P<0.001, ****P<0.0001 (Student's *t*-test, two-tailed). Source data are provided as a Source Data file.

```
RppS  GGGTTCGGAGAAATGAATTATAGCGGCCTCGCGGACCGTCCGGACCAGGCGGGCGGACCGTCCGCGACTGGCTCTGTCTGACATCTGACGACGCATTAAATGCAATATAGCCGTTGATAT 120
RppK  GGGTTCGGAGAAATGAATTATAGCGGCCTCGCGGACCGTCCGGACCAGGCGGGCGGACCGTCCGCGACTGGCTCTGTCTGACATCTGACGACGCATTAAATGCAATATAGCCGTTGATAT 120

RppS  AGCCGTTACTGCTGACCGTTGCATTTTCAGCCGTTGATCTACAGGGGCGGACCGTCCGCACCAGGAGGGCGGACCGTCCGCGCTCAGCAGAATGGCCCAACGGCTAGGAAGTGGTTGGTG 240
RppK  AGCCGTTACTGCTGACCGTTGCATTTTCAGCCGTTGATCTACAGGGGCGGACCGTCCGCACCAGGAGGGCGGACCGTCCGCGCTCAGCAGAATGGCCCAACGGCTAGGAAGTGGTTGGTG 240

RppS  GCTATAAATACAACCCCAACCACCTCCATTCAATCCATCCAAGCATTCCAACCTTCAACATTCAATACAAGAGCTAGCAATCCATTCCAAGACACATTCAAAGCCTCCATCTCTCCAAGT 360
RppK  GCTATAAATACAACCCCAACCACCTCCATTCAATCCATCCAAGCATTCCAACCTTCAACATTCAATACAAGAGCTAGCAATCCATTCCAAGACACATTCAAAGCCTCCATCTCTCCAAGT 360

RppS  TTCACAATTGAGAAAAGAGATCATTAGTGATTAGTAGCTTGAGAGAGAAAGTGATCCGTGTGTTATTTGTCGCTCTTGTTGCTTGGCTTTTTAATCGTGCTTTCTTGATTCTTTCATTGC 480
RppK  TTCACAATTGAGAAAAGAGATCATTAGTGATTAGTAGCTTGAGAGAGAAAGTGATCCGTGTGTTATTTGTCGCTCTTGTTGCTTGGCTTTTTAATCGTGCTTTCTTGATTCTTTCATTGC 480

RppS  GATCAAACTCACTTGTAATTGAGGCAAGAGACACCAATCTTGTGGTGATCCTTGTAGGAACTTTGTGTTCCAAGTGATTGAGAAAAGAAAGCTCACTCGATCCGTGGATCGTTTGAGAGA 600
RppK  GATCAAACTCACTTGTAATTGAGGCAAGAGACACCAATCTTGTGGTGATCCTTGTAGGAACTTTGTGTTCCAAGTGATTGAGAAAAGAAAGCTCACTCGATCCGTGGATCGTTTGAGAGA 600

RppS  GGGAAGGGTTGAAAGAGACCCGGCCTTTGTGGCCTCCTCAACGGGGAGTAGGTTTGCGAGAACCGAACCTCGGTAAAACAAATCCGCGTGTCTCACTTACTTATTCGCTTGCGATTTGTT 720
RppK  GGGAAGGGTTGAAAGAGACCCGGCCTTTGTGGCCTCCTCAACGGGGAGTAGGTTTGCGAGAACCGAACCTCGGTAAAACAAATCCGCGTGTCTCACTTACTTATTCGCTTGCGATTTGTT 720

RppS  TTGCACCCTCTCTCGCGGACTCATTTATTATTACTAACGCTAACCTCGGCTTGTAGTTGTGATTATTTTTGTAAATTTCAGTTTCGCCCTATTCACCCCCCCCTCTAGGCGACTTTCACT 840
RppK  TTGCACCCTCTCTCGCGGACTCATTTATTATTACTAACGCTAACCTCGGCTTGTAGTTGTGATTATTTTTGTAAATTTCAGTTTCGCCCTATTCACCCCCCCCTCTAGGCGACTTTCACT 840

RppS  CACCAGTATTGATTTAAGAACTAAGCGCCTTGTTATTACTGGTCTATTTGGATAAGTAGGAATGACTTAGTTTTTAATAATATTTCAAGTTTACTTATCTGCAGGTTTTTTTTCCAGAG 960
RppK  CACCAGTATTGATTTAAGAACTAAGCGCCTTGTTATTACTGGTCTATTTGGATAAGTAGGAATGACTTAGTTTTTAATAATATTTCAAGTTTACTTATCTGCAGGTTTTTTTTCCAGAG 960

RppS  GTACACACTGGCTTAGATTCTGGGCTCAACTACAAAAGGACGAAGCTGATGGAGTTTTAATAAAGGATGTTTGCCGTCGCCGGGAATCGGTGGTCATGCAATTTTGTGTTAATTTTGGTT 1080
RppK  GTACACACTGGCTTAGATTCTGGGCTCAACTACAAAAGGACGAAGCTGATGGAGTTTTAATAAAGGATGTTTGCCGTCGCCGGGAATCGGTGGTCATGCAATTTTGTGTTAATTTTGGTT 1080

RppS  GGAGGTTTTCTAATAGGATTCCCTTTTAATCATCTTGGTGGTTAAAAAAAAATCAAAAACCTTTAGTGTGCTATGTCCCTTCAGAGAGCAGTGTAATATAACATTTTGTGTTGTCTCTAA 1200
RppK  GGAGGTTTTCTAATAGGATTCCCTTTTAATCATCTTGGTGGTTAAAAAAAAATCAAAAACCTTTAGTGTGCTATGTCCCTTCAGAGAGCAGTGTAATATAACATTTTGTGTTGTCTCTAA 1200

RppS  TCTATTTTGAGGAGAAAGCCGGAACTTTTTCTCGTTATCTGAAAAAATCTTTGGCTAGCCTTCGCGACTCGCCATCTCCCAGCCGTGGCGCTCTCTCGCCCACGCACACCATGCAGCTGC 1320
RppK  TCTATTTTGAGGAGAAAGCCGGAACTTTTTCTCGTTATCTGAAAAAATCTTTGGCTAGCCTTCGCGACTCGCCATCTCCCAGCCGTGGCGCTCTCTCGCCCACGCACACCATGCAGCTGC 1320

RppS  TTCGTCATTTCCAGCCATATGCGTGAAGACATTTGAATTCGTCCACCACTGCATCACTAGTCTCACTCTCACCTATAATATATCTGCACCCAACGAGCAGGTACCAAGCAATTAGCAAGA 1440
RppK  TTCGTCATTTCCAGCCATATGCGTGAAGACATTTGAATTCGTCCACCACTGCATCACTAGTCTCACTCTCACCTATAATATATCTGCACCCAACGAGCAGGTACCAAGCAATTAGCAAGA 1440

RppS  GAAACACAAGGTCGAGGTGGTGAGGCATGGAGCTCGCCTTGGGGGCCATGACCAGCTTGGCCCCTAAGCTTGGCGACCTGCTCATGGAGAAGTATGTCGTGCAGAAGGGCCTCAAGCCCG 1560
RppK  GAAACACAAGGTCGAGGTGGTGAGGCATGGAGCTCGCCTTGGGGGCCATGACCAGCTTGGCCCCTAAGCTTGGCGACCTGCTCATGGAGAAGTATGTCGTGCAGAAGGGCCTCAAGCCCG 1560

RppS  ACATCGAGTCTCTCTCCAGGGAGCTTGTGATGATGAACGCCGCTCTCGTCGACGCGTCCCGGGTTCCACCTGACCAGCTCACCGAGGTGGAAAAGCTCTGGGCACGCAAGGTCCGGGACT 1680
RppK  ACATCGAGTCTCTCTCCAGGGAGCTTGTGATGATGAACGCCGCTCTCGTCGACGCGTCCCGGGTTCCACCTGACCAGCTCACCGAGGTGGAAAAGCTCTGGGCACGCAAGGTCCGGGACT 1680

RppS  TGTCGTATGACATGGAGGACGCCGTCGACGATTTCATCCTGCGTGTGGCTGGTGGTGACGACTCTGCCGCCGACTCCAAATTCTTCAAGAAGACCCTTGCCATGGTCAAGGACGTGATGT 1800
RppK  TGTCGTATGACATGGAGGACGCCGTCGACGATTTCATCCTGCGTGTGGCTGGTGGTGACGACTCTGCCGCCGACTCCAAATTCTTCAAGAAGACCCTTGCCATGGTCAAGGACGTGATGT 1800

RppS  CGATGAAGAAGTTCAAGGATCGGTGCCAGATCTCCGACAAGGTCAAAGACATCAAGAAACTCTCCAACGAGTTAGCTGAACTTCGTGCCAAGTACACGGTAAGGGGTGTGGGTGCTGATC 1920
RppK  CGATGAAGAAGTTCAAGGATCGGTGCCAGATCTCCGACAAGGTCAAAGACATCAAGAAACTCTCCAACGAGTTAGCTGAACTTCGTGCCAAGTACACGGTAAGGGGTGTGGGTGCTGATC 1920

RppS  TCGCCGCGAGCACCGGCATCGACACCCGTGTCATCAATCTGTACAAGAAAGAGACAGATCTCGTTGGTATCGAGGAGTCAAGGGACAAAGTCATTAGGATGCTGTCTATAGGGGCCAAAG 2040
RppK  TCGCCGCGAGCACCGGCATCGACACCCGTGTCATCAATCTGTACAAGAAAGAGACAGATCTCGTTGGTATCGAGGAGTCAAGGGACAAAGTCATTAGGATGCTGTCTATAGGGGCCAAAG 2040

RppS  ATGAAGATGCACATGAGTTCCATCAGGATCTAAAGATAGTGTCTATAGTCGGGGTTGGAGGACTAGGTAAGACTACTCTAGCCAAAACAGTGCATGACATGCTTAAGAAGCAATTCGACT 2160
RppK  ATGAAGATGCACATGAGTTCCATCAGGATCTAAAGATAGTGTCTATAGTCGGGGTTGGAGGACTAGGTAAGACTACTCTAGCCAAAACAGTGCATGACATGCTTAAGAAGCAATTCGACT 2160

RppS  GTTGTGCTTTTATTTCTATTGGTAGAACTCCTAATCTGAATAGGACATTCGAGAAGATGCTATTGGAACTCGATCGTGAGTATAAACAAGTTGACATGGCCAGATGGGATCTAGAACAAT 2280
RppK  GTTGTGCTTTTATTTCTATTGGTAGAACTCCTAATCTGAATAGGACATTCGAGAAGATGCTATTGGAACTCGATCGTGAGTATAAACAAGTTGACATGGCCAGATGGGATCTAGAACAAT 2280

RppS  TTATAAACGAACTGGATGAATTCTTGAAGGACAAGAGGTACGCCCATGTCATTCATTCTCCCTCTAGTTTAGTTGAATTTTTTCATTCACTAGTAGAGCGTGGGCAGTTTTGCCATGTTT 2400
RppK  TTATAAACGAACTGGATGAATTCTTGAAGGACAAGAGGTACGCCCATGTCATTCATTCTCCCTCTAGTTTAGTTGAATTTTTTCATTCACTAGTAGAGCGTGGGCAGTTTTGCCATGTTT 2400

RppS  ATATGCATACCGATTTGCCAAGTTATTCATAGCTGTACACTCTTGTGTCAATTTTATATATATATATATATATATAGGTACTTGATCGTTGTTGATGACATATGGGATGTTGACTCATGGGA 2520
RppK  ATATGCATACCGATTTGCCAAGTTATTCATAGCTGTACACTCTTGTGTCAATTTTATATATATATATATATATATAGGTACTTGATCGTTGTTGATGACATATGGGATGTTGACTCATGGGA 2520

RppS  AGCGATCAAATATGCCTTAAAGGACAATAGTTGTGGAAGTAGAATAATCATGACTACTCGCAATTCTGGGTTTGTCAAGAAAGTAGAAGAGGTTTATAGATTAAAACCTCTTTCTAATGA 2640
RppK  AGCGATCAAATATGCCTTAAAGGACAATAGTTGTGGAAGTAGAATAATCATGACTACTCGCAATTCTGGGTTTGTCAAGAAAGTAGAAGAGGTTTATAGATTAAAACCTCTTTCTAATGA 2640

RppS  AAACTCCAAGAAACTGTTCTACAAAAGAATAGAGAGTCAGGAAGGAGAAAGCCTTGATGGTGAACTCTCTAGTAAAATCATACATAAATGTGGCGGCATACCATTGGCTATCATTGCAAT 2760
RppK  AAACTCCAAGAAACTGTTCTACAAAAGAATAGAGAGTCAGGAAGGAGAAAGCCTTGATGGTGAACTCTCTAGTAAAATCATACATAAATGTGGCGGCATACCATTGGCTATCATTGCAAT 2760

RppS  AGCTAGTTTGTTGGTTGAAAGATCAAGTGAGGAGTGGTCAGAAGTGTACGACAAGATTGGTCTTGGGAATGAGGACAATACAACAAAGATAATGTTATACAGCTACTATGATCTGCCTCC 2880
RppK  AGCTAGTTTGTTGGTTGAAAGATCAAGTGAGGAGTGGTCAGAAGTGTACGACAAGATTGGTCTTGGGAATGAGGACAATACAACAAAGATAATGTTATACAGCTACTATGATCTGCCTCC 2880

RppS  TTATCTCAAGCCATGTCTGCTGCAACTAAGCATATATCCAGAAGACTGTTTCATTGATACAAAAGCTACCATATGGAAGTGGATAGGTGAAGGTCTAGTTCATATTGAGAAAGAGGAGGG 3000
RppK  TTATCTCAAGCCATGTCTGCTGCAACTAAGCATATATCCAGAAGACTGTTTCATTGATACAAAAGCTACCATATGGAAGTGGATAGGTGAAGGTCTAGTTCATATTGAGAAAGAGGAGGG 3000

RppS  TAGCCTATTTAAGGTTGGAGAAAGATACTTCAAGGAGCTTGTGAATAGAAGCATGATCCAGCCGATAGAGAACATAAATGATTGGTTTGTAGAAGAGTTCCGTATTCACGACATTGTGTT 3120
RppK  TAGCCTATTTAAGGTTGGAGAAAGATACTTCAAGGAGCTTGTGAATAGAAGCATGATCCAGCCGATAGAGAACATAAATGATTGGTTTGTAGAAGAGTTCCGTATTCACGACATTGTGTT 3120

RppS  TGATCTCATCTGTAAGTTGTCCAAGGATGAAGACTTCATTAGCCTTAGCGGGCAACATTCATCTCAGGATAGTTTAAGAAGAGAGAAGAAAACAGGTGTGCCTCGCTCAGACTGCAAGCT 3240
RppK  TGATCTCATCTGTAAGTTGTCCAAGGATGAAGACTTCATTAGCCTTAGCGGGCAACATTCATCTCAGGATAGTTTAAGAAGAGAGAAGAAAACAGGTGTGCCTCGCTCAGACTGCAAGCT 3240

RppS  ACGTCGTCTGGTCGTCCGAAATCAACGTGTGCAGCGCTTCCCTGAAGAAACCATGGACATGCCAGAGGTGTTGAGATCACTTAGCATTATAGATTGTAATATTGCGGTTGTGGCCCCAAT 3360
RppK  ACGTCGTCTGGTCGTCCGAAATCAACGTGTGCAGCGCTTCCCTGAAGAAACCATGGACATGCCAGAGGTGTTGAGATCACTTAGCATTATAGATTGTAATATTGCGGTTGTGGCCCCAAT 3360

RppS  TGATAGCTTCAGGGTTTGCCGTGTGCTGTCTATAGTAAACAACTACGTACCCATCAGCCTAAAGCATCTGGGGAAGCTGTTGCATCTCAAGTTCCTAGAGATAGTATACACGCCTATTGA 3480
RppK  TGATAGCTTCAGGGTTTGCCGTGTGCTGTCTATAGTAAACAACTACGTACCCATCAGCCTAAAGCATCTGGGGAAGCTGTTGCATCTCAAGTTCCTAGAGATAGTATACACGCCTATTGA 3480

RppS  TGAGCTCCCTAAGGAAATTGGGCATCTGAGGTCTCTTCAGACACTGATATTAGTCCGTACTGGACTAGACGAGCTGCCACCGGCTCTTTGCTCGCTTACACGGCTCATGTGTCTGATAGC 3600
RppK  TGAGCTCCCTAAGGAAATTGGGCATCTGAGGTCTCTTCAGACACTGATATTAGTCCGTACTGGACTAGACGAGCTGCCACCGGCTCTTTGCTCGCTTACACGGCTCATGTGTCTGATAGC 3600

RppS  CTATGGCTTCGAAAGGTTGCCAGCTGATAGGATGGGGAACCTAACGTCCCTGGAGGAGCTACAACTAAATAGGGTAGTTGGCCGGAGTGCCACCCAAGACCTAGTGGCAGAGTTTGGCAA 3720
RppK  CTATGGCTTCGAAAGGTTGCCAGCTGATAGGATGGGGAACCTAACGTCCCTGGAGGAGCTACAACTAAATAGGGTAGTTGGCCGGAGTGCCACCCAAGACCTAGTGGCAGAGTTTGGCAA 3720

RppS  GCTGACGAGGTTGAGGGTGGTCAGCATCACCTTTTCAGAGCAGCTAGAGGAGAGCTTGCAAGAAGCATTGGTGCAATCTCTGTCCAATCTGCGGCGACTCCAGGAACTAGAGCTTTTGTG 3840
RppK  GCTGACGAGGTTGAGGGTGGTCAGCATCACCTTTTCAGAGCAGCTAGAGGAGAGCTTGCAAGAAGCATTGGTGCAATCTCTGTCCAATCTGCGGCGACTCCAGGAACTAGAGCTTTTGTG 3840

RppS  TAAAATGCCAGAGCGGGGAAGCGATATGTGGGGAGACTGGGAGCCACCAAGGCAGCTCCGGCGCCTGATTATTGAAGGCATCGACTTCTCACGGCAGCCTCGATGGATCAACCGCTCCTG 3960
RppK  TAAAATGCCAGAGCGGGGAAGCGATATGTGGGGAGACTGGGAGCCACCAAGGCAGCTCCGGCGCCTGATTATTGAAGGCATCGACTTCTCACGGCAGCCTCGATGGATCAACCGCTCCTG 3960

RppS  CCTGCCACGCCTCTGCTCCTTATATCTGAGGGTGCACGCTCTTGAAGCACAGGACCTAGATAATCTAGCGAGGTTGCCAGAGCTCCAGTACCTCCAGCTATTTGGTCTCAGCTTTCCTCC 4080
RppK  CCTGCCACGCCTCTGCTCCTTATATCTGAGGGTGCACGCTCTTGAAGCACAGGACCTAGATAATCTAGCGAGGTTGCCAGAGCTCCAGTACCTCCAGCTATTTGGTCTCAGCTTTCCTCC 4080

RppS  AAGGTATACTGTTGGCCCAGACGACTTCAGGAATCTGAGGTTCTGCGAAGTGGGCACAACGTTCGAGTTTCGTAAGGGCGCCATGCCAAGGCTTGAAGTGCTGCGATTTGGAGTTTATGC 4200
RppK  AAGGTATACTGTTGGCCCAGACGACTTCAGGAATCTGAGGTTCTGCGAAGTGGGCACAACGTTCGAGTTTCGTAAGGGCGCCATGCCAAGGCTTGAAGTGCTGCGATTTGGAGTTTATGC 4200

RppS  AGGGTACTGGAGTTGGGAAGAGAATGGTGTGCCGTTCGAGCAGTTCCCAACGAAGGATGTGATCGAAGATCTTCACTTGGACCTGGATAACGTCCTTTTACTTCAGCAAGTAATAGTCAA 4320
RppK  AGGGTACTGGAGTTGGGAAGAGAATGGTGTGCCGTTCGAGCAGTTCCCAACGAAGGATGTGATCGAAGATCTTCACTTGGACCTGGATAACGTCCTTTTACTTCAGCAAGTAATAGTCAA 4320

RppS  AGTCAACTGCTTAGGTGCTACTGCCGCACAAGTGGAGGAGGTGGAGGCCGTGGTCATGCGTGCCGTGGAAAATCATGCCAACCGTCCAACCATAAAAATGGATCGAGTATATGAAGAAAA 4440
RppK  AGTCAACTGCTTAGGTGCTACTGCCGCACAAGTGGAGGAGGTGGAGGCCGTGGTCATGCGTGCCGTGGAAAATCATGCCAACCGTCCAACCATAAAAATGGATCGAGTATATGAAGAAAA 4440

RppS  TATCTTATCTGATGAAAAGTGGGAGGCTCTGGTGAGTCTGCTAAAGAAAATATGTTTTCTTATATATATTTATATATATATATATATATAT--TTATGTTTGTGCACTTCAATTCTCAGC 4558
RppK  TATCTTATCTGATGAAAAGTGGGAGGCTCTGGTGAGTCTGCTAAAGAAAATATGTTTTCTTATATATATTTATATATATATATATATATATATTTATGTTTGTGCACTTCAATTCTCAGC 4560

RppS  TTCGGCGACACATTGAAGAGGATTGCTGCGTGCGCACGATGAAGGATAAATCTAATGCTTTCTTCATCAGCCAGCTGTGGTTATATCGACATCTTCAGGAAGCCATTATTTTCATCGACT 4678
RppK  TTCGGCGACACATTGAAGAGGATTGCTGCGTGCGCACGATGAAGGATAAATCTAATGCTTTCTTCATCAGCCAGCTGTGGTTATATCGACATCTTCAGGAAGCCATTATTTTCATCGACT 4680

RppS  GTTCGGGTGCCAGCATGTGTGAGGTGCAGAAAGTGGAAGCAGCTTATAGACATGCAGCCGAGGTTCATCCTAACCATCCAAGTATTGAACTTATCAGAACAAACACCGACGGAATGGCCT 4798
RppK  GTTCGGGTGCCAGCATGTGTGAGGTGCAGAAAGTGGAAGCAGCTTATAGACATGCAGCCGAGGTTCATCCTAACCATCCAAGTATTGAACTTATCAGAACAAACACCGACGGAATGGCCT 4800

RppS  CCTCCTCATCTGACCATCCCAACACAGAGGTTCGTCCTAGCTGCGTGGAAAATTTTTCAGTAATTAATGGAAACCTTAGCTTTGACGGTATAACTTACCACCAACGCTTATTTTTCGTTC 4918
RppK  CCTCCTCATCTGACCATCCCAACACAGAGGTTCGTCCTAGCTGCGTGGAAAATTTTTCAGTAATTAATGGAAACCTTAGCTTTGACGGTATAACTTACCACCAACGCTTATTTTTCGTTC 4920

RppS  CCAAACTCCAGCCCAGGAATTGATGACTCGGTTTGTCAGATCCTTCGACCCAGCACCAGCAGCAGGTACGTGTGCATGCCCATGATCTACACTCTTAAATTAATTTTATTACTCTATCCA 5038
RppK  CCAAACTCCAGCCCAGGAATTGATGACTCGGTTTGTCAGATCCTTCGACCCAGCACCAGCAGCAGGTACGTGTGCATGCCCATGATCTACACTCTTAAATTAATTTTATTACTCTATCCA 5040

RppS  GTTATCACGTTCCAATGCCATG 5060
RppK  GTTATCACGTTCCAATGCCATG 5062
```

Supplementary Figure 17. Nucleotide sequence alignment between *RppS* and *RppK.* The *RppS* sequence from the inbred line SCML205 was amplified with primers R8.64F and R8.63R (Supplementary Data 5). The difference between the two sequences, highlighted with a yellow background, is located in the 2nd intron of the *R3* gene. Primer sequences are underlined. The sequences of four exons are shown in red. The start codons (ATG) and stop codons (TGA) are underlined in red.

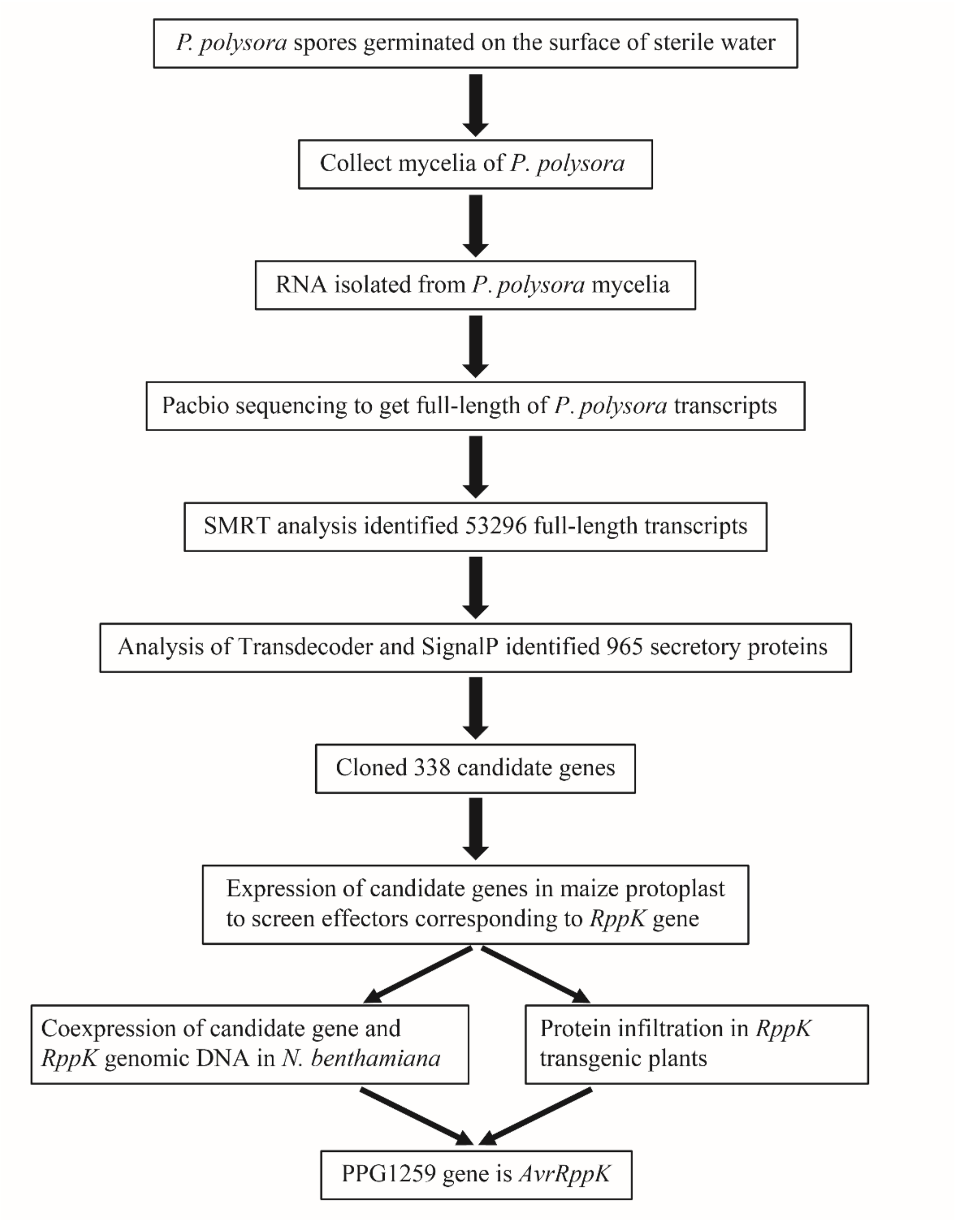

Supplementary Figure 18. The pipeline for *AvrRppK* gene screening. To obtain the mRNA sequences of *P. polysora* secretory genes, the urediospores of *P. polysora* were germinated on the water surface, and the collected mycelia were used for mRNA isolation. Then, mRNA was sequenced by PacBio sequencing. Through SMRT v2.3, Transdecoder v4.1.0 and SignalP-4.0 analysis, 965 genes were predicted to encode secretory proteins, and 338 of these genes were successfully cloned. In transient expression assays in the protoplasts of transgenic *RppK* plants and nontransgenic control plants, only *PPG1259* induced an HR in protoplasts of transgenic *RppK* plants and not in those of nontransgenic control plants. The coexpression of *PPG1259* with *RppK* genomic DNA in *N. benthamiana* induced an HR; the infiltration of purified PPG1259 protein induced an HR in transgenic *RppK* plants but not in nontransgenic control plants. All these results confirmed that *PPG1259* is the *AvrRppK* gene.

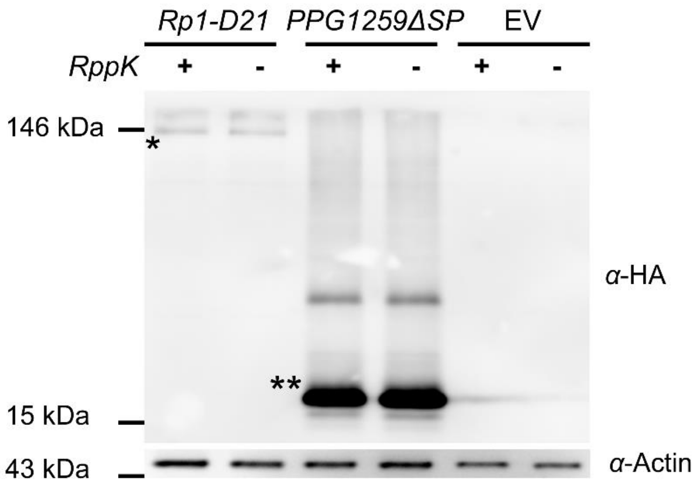

Supplementary Figure 19. Western blot to detect the protein levels of Rp1-D21 and PPG1259ΔSP. After *RP1-D21-3×HA*, *PPG1259ΔSP-3×HA* and empty vector (EV) were transiently expressed with *35s:LUC* construct in protoplasts of *RppK* transgenic positive plants and *RppK* transgenic negative plants. 16 hours after transformation, proteins were isolated and were examined by western blot using anti-HA antibody (dilution 1:1000). The maize Actin protein was taken as the loading control, which was examined by western blot using anti-Actin antibody (dilution 1:5000). Rp1-D21-3×HA protein was labelled with “*” and PPG1259ΔSP-3×HA was labelled with “**”. “+” means protoplasts from *RppK* transgenic positive plants and “-” means protoplasts from *RppK* transgenic negative plants. The experiment was repeated three times with similar results. Source data are provided as a Source Data file.

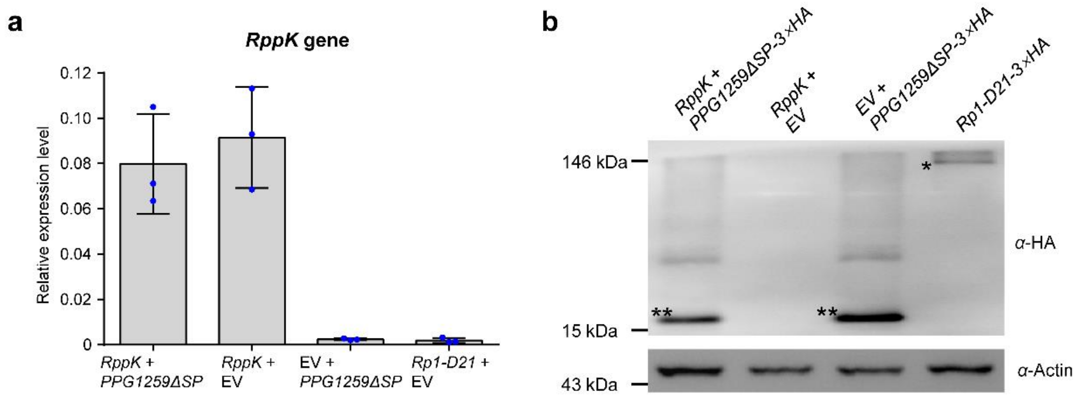

Supplementary Figure 20. Evaluation of gene expression levels and protein levels in *N. benthamiana* transient expression assay. (a) Detection of *RppK* gene expression by RT-qPCR at 3 days after *RppK* genomic DNA construct was co-infiltrated into *N. benthamiana* with *PPG1259ΔSP-3×HA* or empty vector (EV) or empty vector was co-infiltrated into *N. benthamiana* with *PPG1259ΔSP-3×HA* or *Rp1-D21*. Values are means ± SDs, n=3 repeats. The experiment was repeated three times with similar results. **(b)** Detection of protein levels of PPG1259ΔSP-3×HA and Rp1-D21-3×HA by western blot at 3 days after *RppK* genomic DNA construct was co-infiltrated into *N. benthamiana* with *PPG1259ΔSP-3×HA* or empty vector (EV) or empty vector was co-infiltrated into *N. benthamiana* with *PPG1259ΔSP-3×HA* or *Rp1-D21*. Rp1-D21-3×HA protein was labelled with one "*" and PPG1259ΔSP-3×HA was labelled with "**". The experiment was repeated three times with similar results. Source data are provided as a Source Data file.

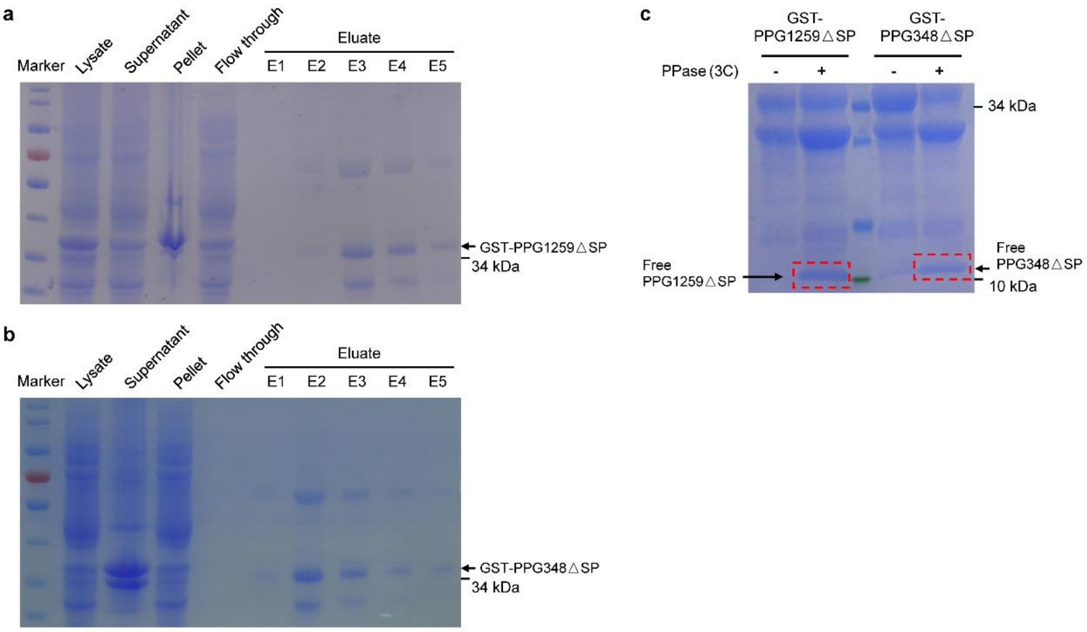

Supplementary Figure 21. The expression and purification of GST-PPG1259ΔSP and GST-PPG348ΔSP in *E. coli*. (a) The expression and purification of GST-PPG1259ΔSP. **(b)** The expression and purification of GST-PPG348ΔSP. **(c)** Deletion of GST tags of recombinant proteins by 3C PPase. Purified GST-PPG1259ΔSP and GST-PPG348ΔSP proteins were treated with 3C PPase to remove the GST tag. E1-E5 were different eluate collected in different tubes. Source data are provided as a Source Data file.

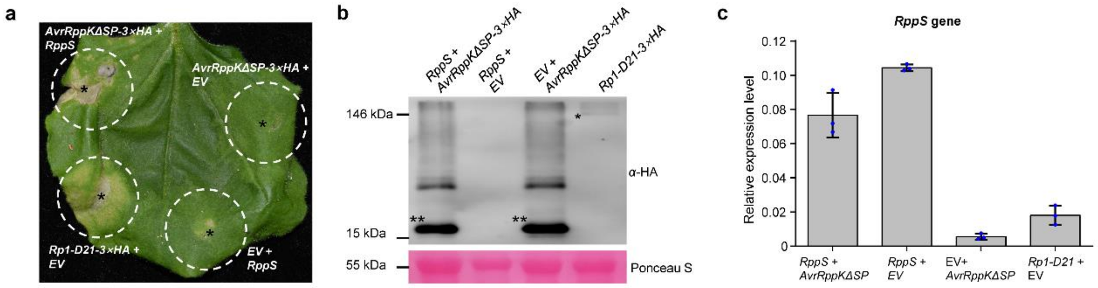

Supplementary Figure 22. Coexpression of *RppS* and *AvrRppK* induces HR in *N. benthamiana*. (a) The coinfiltration of the construct carrying the *RppS* genomic DNA sequence with the *AvrRppKΔSP-3×HA* construct induced an HR in *N. benthamiana*. The coinfiltration of the construct carrying the *RppS* genomic DNA construct or the *AvrRppKΔSP* construct with the empty vector (EV) were performed as negative controls and the infiltration of *Rp1-D21-3×HA* was taken as the positive control. The infiltration sites were labeled with "*". **(b)** Western blot to check the protein levels of AvrRppKΔSP-3×HA and Rp1-D21-3×HA at three days after infiltration. Rp1-D21-3×HA protein was labelled with "*" and AvrRppKΔSP-3×HA protein was labelled with "**". **(c)** RT-qPCR to check the transcription level of *RppS* at three days after infiltration. Values are means ± SDs, n=3. Source data are provided as a Source Data file.

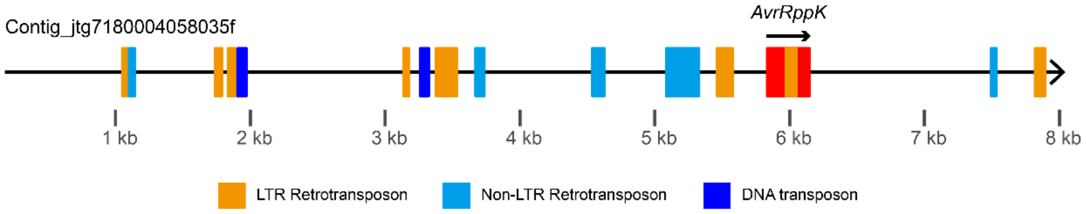

Supplementary Figure 23. The *AvrRppK* gene is surrounded by many repeat sequences or transposons. The repeat sequences are annotated and indicated with different colors. The *AvrRppK* gene, located in contig_jtg7180004058035f, is indicated with a red box and contains a transposon. Orange, LTR retrotransposon. Sky blue, Non-LTR retrotransposon. Dark blue, DNA transposon.

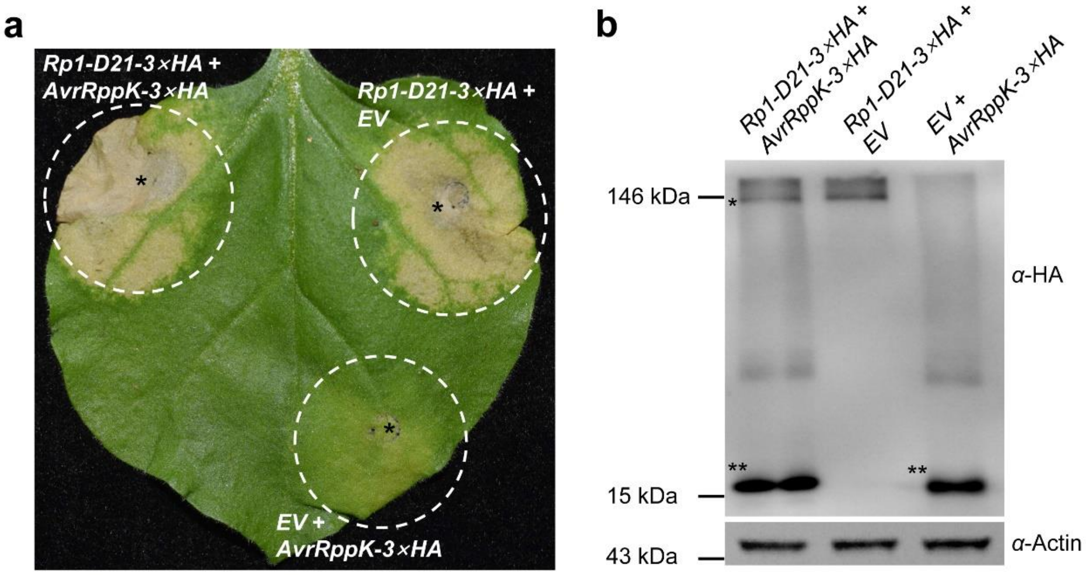

Supplementary Figure 24. Expression of *AvrRppK* cannot suppress *Rp1-D21*-mediated HR. (a) Coinfiltration of *Rp1-D21-3×HA* with *AvrRppKΔSP-3×HA* in *N. benthamiana* showed cell death phenotype at three days after infiltration. Coinfiltration of *Rp1-D21-3×HA* with empty vector (EV) was taken as the positive control. The infiltration sites were labelled with "*". **(b)** Western blot to check the protein levels of Rp1-D21-3×HA and AvrRppKΔSP-3×HA by anti-HA antibody (dilution 1:1000). Rp1-D21-3×HA protein was labelled with "*" and AvrRppKΔSP-3×HA was labelled with "**". The protein level of *N. benthamiana* Actin protein was taken as the loading control, which was examined by western blot using anti-Actin antibody (dilution 1:5000). The experiment was repeated three times with similar results. Source data are provided as a Source Data file.

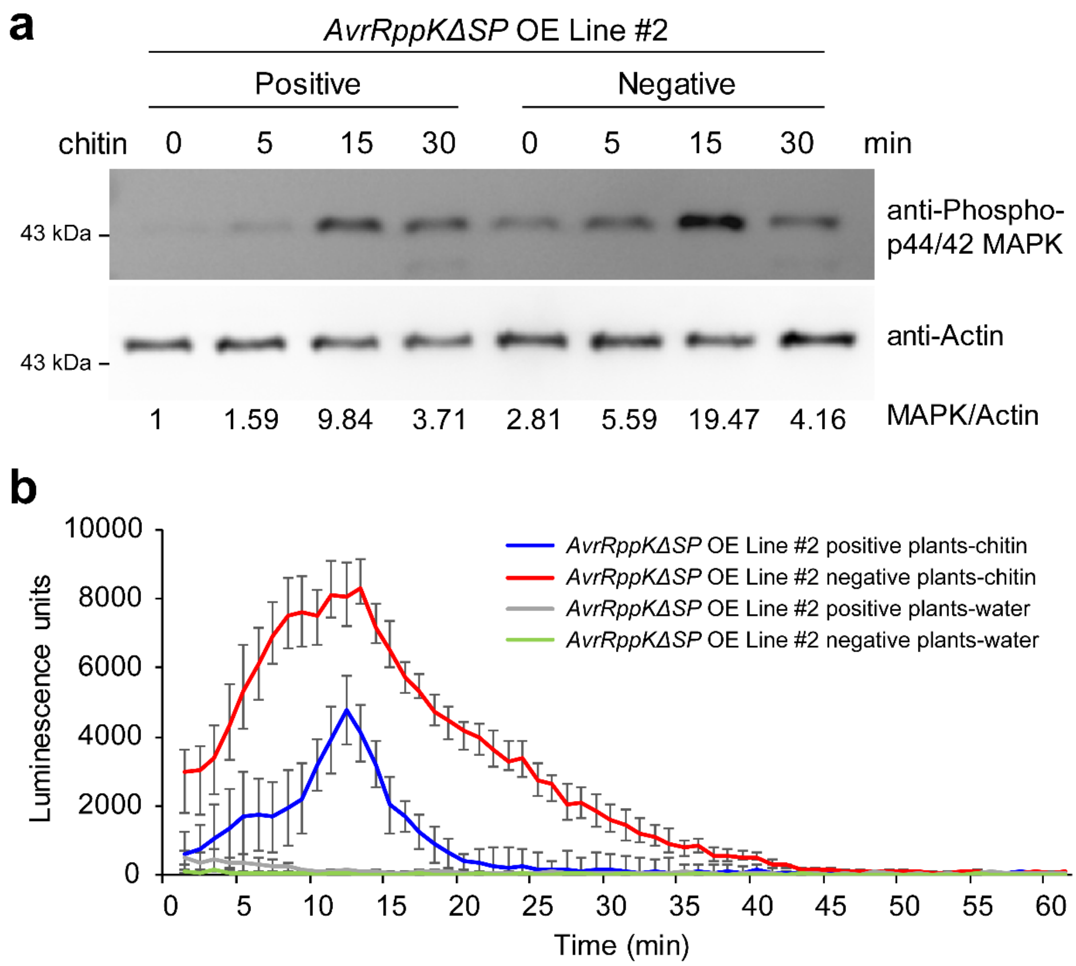

Supplementary Figure 25. AvrRppK suppresses chitin-triggered PTI. (a) The chitin-triggered MAP kinase activity was suppressed by *AvrRppK*. Positive and negative plants in *AvrRppKΔSP* overexpression (OE) transgenic maize line#2 were treated with chitin at different time points (0, 5, 15 and 30 minutes). Western blot was used to detect MAP kinase activity by anti-Phospho-p44/42 MAPK antibody (dilution 1:1000). The loading control was maize Actin proteins measured by western blot using anti-Actin antibody (dilution 1:5000). Relative MAP kinase activity was normalized to the level of maize Actin protein. **(b)** Chitin-induced ROS accumulation was suppressed by *AvrRppK*. Positive and negative plants in *AvrRppKΔSP* overexpression transgenic maize line#2 were treated with chitin at different time points (from 1 to 60 minutes). The absolute luminescence was used to represent ROS accumulation. Water treatment was taken as the control. Values are means ± SDs, n=3 repeats. Source data are provided as a Source Data file.

Supplementary Table 1. Summary of QTL mapping for southern corn rust in KD RIL population by CIM method.

Population	Chromosome	Left marker	Right marker	QTL interval (cM)	LOD	Phenoype variance explained (%)	Additive effect	QTL
DAN340/K22 RILs	8	PZE-108092412	PZE-108092671	96-96.4	5.1	2.7	-0.2	
	10	SYN16185	SYN16808	3.3-4.4	48.8	68	-1.1	*RppK*

Supplementary Table 2. Haplotype analysis of the *R3* gene in association mapping panel.

Group	Haplotype	Marker[#]				Population[&]			Hap number	SCR score[§]
		Del13K	R8.65	R8.63	R8.61[$]	Tropical	Temperate	Mixed		
Group I	5213-like	-	-	-	Hap-I	19	12	18	49	3.62±1.74
	303WX-like	-	-	-	Hap-II	16	7	8	31	2.97±1.24
	1323-like	-	-	-	Hap-III	36	2	7	45	2.57±1.16
	B73-like	-	-	-	Hap-IV	80	74	29	183	3.17±1.37
	Gy237-like	-	-	-	Hap-V	1	2	2	5	4.59±1.85
	Jiao51-like	-	-	-	Hapmixed	17	9	13	39	3.45±1.59
Group II	1145-like	DR3	-	-	Hap-I	3	4	5	12	3.11±1.52
	B77-like	DR3	-	-	Hap-III	1	2	2	5	3.22±0.96
	DAN340-like	DR3	-	-	Hap-IV	12	25	8	45	3.99±1.35
	TY9-like	DR3	-	-	-	2	7	4	13	4.1±1.24
	Sy1032-like	DR3	-	-	Hap-V	0	0	1	1	5.11
	Zheng30-like	DR3	-	-	Hapmixed	0	2	3	5	5.43±1.11
Group III	CIMBL63-like	-	-	R3	-	1	0	0	1	1.37
	CML433-like	-	R2	R3	-	1	1	0	2	2.29±1.67
	K22-like	-	R1R2	R3	-	5	4	3	12	1.99±0.8
	1462-like	-	R1R2	R3	Hap-III	0	1	0	1	4.39
	CIMBL35-like	-	R1R2	R3	Hap-IV	1	0	0	1	1.07
Group IV	None-R-like	-	-	-	-	24	11	14	49	2.95±1.58
	Total					219	163	117	499	

[#] molecular markers used to genotype different maize inbred lines. "-" stands for no PCR fragment amplified. The haplotypes ("R1R2" or "R2") of R8.65 are distinguished by sequencing the R8.65 PCR products according to Supplementary Figure 10c, "R1R2" indicates the genotype contains both of R1 and R2 genes, "R2" indicates the genotype only contains R2 gene.

[$] "Hapmixed" indicates that it contains multiple classes of R8.61 product.

[&] numbers of inbred lines in each subpopulation.

[§] Values are means ± SD.

Supplementary Table 3. The relationship among *Rpp* genes.

Line	Resistant loci	Del13K[#]	R8.65[#]	R8.63[#]	R8.61[#]	Haplotype of *RppK*	R/S	Reference
K22	*RppK*	-	R1R2	R3	-	K22-like	R	This study
SCML205	*RppS (RppK)*	-	R1R2	R3	-	K22-like	R	1
CML496	*RppC*	-	-	-	Hap-I	5213-like	R	2
QI319	*RppQ*	-	-	-	Hap-IV	B73-like	R	3
P25	*RppP25*	-	-	-	Hap-IV	B73-like	R	4
CML470	*RppCML470*	-	-	-	-	None-R-like	R	5
W2D	*RppD*	na	na	na	na	na	R	6
S313	*RppS313*	na	na	na	na	na	R	7

[#] molecular markers used to genotype different maize inbred lines (Supplementary Fig. 10b-c); "-" stands for no PCR fragment amplified; "na" means that those maize lines were not detected because seeds were not available.

Supplementary Table 4. The information of 17 inbred lines containing *RppK* locus

Line	SCR BLUP	Subpopulation	Pedigree	Origin
K22	1.14	NSS	K11×Ye478	China
526018	1.93	NSS	Unkown	China
975-12	1.7	Mixed	78698×Dan9046	China
CHENG698	4.04	Mixed	Foreign Hybrid	China
CIMBL134	1.34	TST	(SAM4(Angola)/[BETASYN]BC1-79-2-3-B-B)-B	CIMMYT
CIMBL135	1.1	TST	(SAM4(Angola)/[BETASYN]BC1-82-1-2-B-B)-B	CIMMYT
CML432	2.3	TST	(CML- 432)-B	CIMMYT
LXN	2.1	NSS	Landrace	China
M97	2.37	NSS	Unknown	China
SW1611	1.34	TST	Suwan2	China
YUN46	2.26	TST	Yun46	China
Z2018F	2.28	Mixed	Zheng2018F2*698-3-1-2-1-1	China
1462	4.39	NSS	Unkown	China
CIMBL35	1.07	TST	((KU1409/KU1414-SR/KVI43)-S2-4-2-B-B)-B	CIMMYT
CML433	1.11	TST	(CML- 433)-B	CIMMYT
DAN9046	3.47	NSS	Shen5003×Tie7922	China
CIMBL63	1.37	TST	(Carotenoid Syn3-FS5-1-5-B-B)-B	CIMMYT

The information in Supplementary Table 4 was published before[8].

Supplementary references

1. Wu, X. *et al*. Geographic and genetic identification of *RppS*, a novel locus conferring broad resistance to southern corn rust disease in China. *Euphytica* **205**, 17-23 (2015).
2. Lv, M. *et al*. Identification and fine-mapping of *RppCML496*, a major QTL for resistance to *Puccinia polysora* in maize. *Plant Genome* **14**, e20062 (2021).
3. Chen, C. *et al*. Molecular tagging and genetic mapping of the disease resistance gene *RppQ* to southern corn rust. *Theor Appl Genet* **108**, 945-950 (2004).
4. Liu Z. *et al*. Studies of genetic analysis and SSR linked marker location of gene resistance to southern rust in inbred line P25 of maize. *Yi Chuan Xue Bao* **30**, 706-10 (2003).
5. Yao, G. *et al*. Mapping the maize southern rust resistance gene in inbred line CML470. *J Plant Gene Res* **14**, 518-522 (2013).
6. Zhang, Y. *et al*. Mapping of southern corn rust-resistant genes in the W2D inbred line of maize (*Zea mays* L.). *Mol Breeding* **25**, 433-439 (2010).
7. Wang, B. *et al*. QTL mapping and genetic analysis of a gene with high resistance to southern corn rust. *Scientia Agricultura Sinica* **52**, 2033-2041 (2019).
8. Yang, X. *et al*. Characterization of a global germplasm collection and its potential utilization for analysis of complex quantitative traits in maize. *Mol Breeding* **28**, 511-526 (2011).
