# Supplementary material for: Cloning southern corn rust resistant gene RppK and its cognate gene AvrRppK from Puccinia polysora
Source: Nat Commun. 2022 Jul 29;13:4392. doi: 10.1038/s41467-022-32026-4 (PMC9338322; doi:10.1038/s41467-022-32026-4)
Supplement: Supplementary file 3 — Description of Additional Supplementary Files [file 41467_2022_32026_MOESM3_ESM.pdf]

Description of Additional Supporting Information

File name: Supplementary Data 1.
Description: The haplotype of *RppK* loci in 500 inbred lines of the association mapping panel.

File name: Supplementary Data 2.
Description: The information of 288 landrace accessions.

File name: Supplementary Data 3.
Description: The information of 166 teosinte accessions.

File name: Supplementary Data 4.
Description: The information of 74 hybrids commercially cultivated in China.

File name: Supplementary Data 5.
Description: Primers used in this study.
