# Supplementary material for: Cloning southern corn rust resistant gene RppK and its cognate gene AvrRppK from Puccinia polysora
Source: Nat Commun. 2022 Jul 29;13:4392. doi: 10.1038/s41467-022-32026-4 (PMC9338322; doi:10.1038/s41467-022-32026-4)
Supplement: Supplementary file 9 — Reporting Summary [file 41467_2022_32026_MOESM9_ESM.pdf]

nature portfolio

Corresponding author(s): Jianbing Yan and Zhibing Lai

Last updated by author(s): Jun 18, 2022

Reporting Summary

Nature Portfolio wishes to improve the reproducibility of the work that we publish. This form provides structure for consistency and transparency in reporting. For further information on Nature Portfolio policies, see our Editorial Policies and the Editorial Policy Checklist.

Statistics

For all statistical analyses, confirm that the following items are present in the figure legend, table legend, main text, or Methods section.

n/a	Confirmed	
☐	☒	The exact sample size (*n*) for each experimental group/condition, given as a discrete number and unit of measurement
☐	☒	A statement on whether measurements were taken from distinct samples or whether the same sample was measured repeatedly
☐	☒	The statistical test(s) used AND whether they are one- or two-sided<br>*Only common tests should be described solely by name; describe more complex techniques in the Methods section.*
☒	☐	A description of all covariates tested
☐	☒	A description of any assumptions or corrections, such as tests of normality and adjustment for multiple comparisons
☐	☒	A full description of the statistical parameters including central tendency (e.g. means) or other basic estimates (e.g. regression coefficient) AND variation (e.g. standard deviation) or associated estimates of uncertainty (e.g. confidence intervals)
☐	☒	For null hypothesis testing, the test statistic (e.g. *F*, *t*, *r*) with confidence intervals, effect sizes, degrees of freedom and *P* value noted<br>*Give P values as exact values whenever suitable.*
☒	☐	For Bayesian analysis, information on the choice of priors and Markov chain Monte Carlo settings
☒	☐	For hierarchical and complex designs, identification of the appropriate level for tests and full reporting of outcomes
☒	☐	Estimates of effect sizes (e.g. Cohen's *d*, Pearson's *r*), indicating how they were calculated

Our web collection on statistics for biologists contains articles on many of the points above.

Software and code

Policy information about availability of computer code

Data collection	no software was used.
Data analysis	SMRT analysis (V2.3); Transdecoder v4.1.0; GraphPad Prism 8; SignalP-4.0;

For manuscripts utilizing custom algorithms or software that are central to the research but not yet described in published literature, software must be made available to editors and reviewers. We strongly encourage code deposition in a community repository (e.g. GitHub). See the Nature Portfolio guidelines for submitting code & software for further information.

Data

Policy information about availability of data

All manuscripts must include a data availability statement. This statement should provide the following information, where applicable:

- Accession codes, unique identifiers, or web links for publicly available datasets
- A description of any restrictions on data availability
- For clinical datasets or third party data, please ensure that the statement adheres to our policy

The RppK locus genomic DNA sequences from K22, DAN340 and 1145 are available at NCBI under accessions MZ322317, MZ322318, MZ312612; the RNA-seq data generated by PacBio sequencing is available at NCBI under the BioProject ID: PRJNA732947; the P. polysora genomic DNA sequence data generated by single cell is available under the BioProject ID: PRJNA732557.

Field-specific reporting

Please select the one below that is the best fit for your research. If you are not sure, read the appropriate sections before making your selection.

☒ Life sciences ☐ Behavioural & social sciences ☐ Ecological, evolutionary & environmental sciences

For a reference copy of the document with all sections, see nature.com/documents/nr-reporting-summary-flat.pdf

Life sciences study design

All studies must disclose on these points even when the disclosure is negative.

Sample size	The sample size and related analysis methods are described in the figure legends or method section. Sample size were determined based on published papers (Xiao et al., 2016, New Phytopathologist; Zeng, BZ. 1994, Genetics; Yang et al., 2011; Molecular breeding;)
Data exclusions	No data were excluded.
Replication	Each experiment was repeated at least three times with the similar results. and all attempts at replication were successful.
Randomization	plants with different genotypes or transgenic plants with positive or negative genotype were planted side by side to minimize the phenotypic difference under different conditions (in the field) or in the growth room. For disease scale evaluation, all plants were evaluated. For collecting uredia of Puccinia polysora, leaf samples with similar age were randomly picked up for experiments. And Puccinia polysora uredia were randomly collected from the leaf samples collected from the field and only one Puccinia polysora uredium was collected from each leaf sample.
Blinding	Plant breeding, disease phenotype evaluation, genotype identification and data analysis were done by different person. And the investigators were blinded to people for allocation during data collection

Reporting for specific materials, systems and methods

We require information from authors about some types of materials, experimental systems and methods used in many studies. Here, indicate whether each material, system or method listed is relevant to your study. If you are not sure if a list item applies to your research, read the appropriate section before selecting a response.

Materials & experimental systems

n/a	Involved in the study	
☐	☒	Antibodies
☒	☐	Eukaryotic cell lines
☒	☐	Palaeontology and archaeology
☒	☐	Animals and other organisms
☒	☐	Human research participants
☒	☐	Clinical data
☒	☐	Dual use research of concern

Methods

n/a	Involved in the study	
☒	☐	ChIP-seq
☒	☐	Flow cytometry
☒	☐	MRI-based neuroimaging

Antibodies

Antibodies used	anti-HA antibody (Abcom, Cat#ab49969, dilution 1:1,000); anti-Actin antibody (ABclonal, Cat#AC009, dilution 1:5,000);Phospho-p44/42 MAPK (Erk1/2) (Thr202/Tyr204) Antibody (Cell signaling Technology, Cat#9101, dilution 1:1,000)
Validation	1) anti-HA antibody (Abcom, Cat#ab49969): It is species independent. https://www.abcam.com/ha-tag-antibody-ha-7-ab49969.html<br>2) anti-Actin antibody (ABclonal, Cat#AC009): https://abclonal.com.cn/catalog/AC009;<br>3) Phospho-p44/42 MAPK (Erk1/2) (Thr202/Tyr204) Antibody (Cell signaling Technology, Cat#9101): https://www.cellsignal.com/products/primary-antibodies/phospho-p44-42-mapk-erk1-2-thr202-tyr204-antibody/9101?_=1640075747709&Ntt=9101&tahead=true

March 2021
